# KCTD9 inhibits the Wnt/β-catenin pathway by decreasing the level of β-catenin in colorectal cancer

**DOI:** 10.1038/s41419-022-05200-1

**Published:** 2022-09-02

**Authors:** Hanhui Yao, Delong Ren, Yichun Wang, Liang Wu, Yang Wu, Wei Wang, Qidong Li, Lianxin Liu

**Affiliations:** 1 Department of Hepatobiliary Surgery, Anhui Provincial Clinical Research Center for Hepatobiliary Diseases, Anhui Province Key Laboratory of Hepatopancreatobiliary Surgery, The First Affiliated Hospital of USTC, Division of Life Sciences and Medicine, University of Science and Technology of China, Hefei, 230001 China; 2 Department of Medical Oncology, The First Affiliated Hospital of USTC, Division of Life Sciences and Medicine, University of Science and Technology of China, Hefei, 230001 China

**Keywords:** Colon cancer, Epithelial-mesenchymal transition

## Abstract

Colorectal cancer (CRC) is the second leading cause of cancer mortality worldwide. However, the molecular mechanisms underlying CRC progression remain to be further defined to improve patient outcomes. In this study, we found that KCTD9, a member of the potassium channel tetramerization domain-containing (*KCTD*) gene family, was commonly downregulated in CRC tissues and that KCTD9 expression was negatively correlated with the clinical CRC stage. Survival analysis showed that patients whose tumors expressed low KCTD9 levels had poorer outcomes. Functional analyses revealed that KCTD9 overexpression inhibited CRC cell proliferation and metastasis, whereas KCTD9 knockdown promoted CRC cell proliferation and metastasis in both in vitro and in vivo models. Manipulating KCTD9 levels in CRC cells via overexpression or knockdown showed KCTD9 expression positively influenced the degradation of β-catenin levels leading to inhibition of Wnt signaling and reductions in Wnt pathway target gene expression. Mechanistically, we found KCTD9 associated with ZNT9 (Zinc Transporter 9), a coactivator of β-catenin-mediated gene transcription. The overexpression of KCTD9 or knockdown of ZNT9 in CRC cells increased the polyubiquitination and proteasomal degradation of β-catenin. In turn, the KCTD9-ZNT9 interaction disrupted interactions between β-catenin and ZNT9, thereby leading to decreased β-catenin target gene expression and the inhibition of Wnt signaling. In conclusion, our findings propose that KCTD9 functions as a tumor suppressor that inhibits CRC cell proliferation and metastasis by inactivating the Wnt/β-catenin pathway. Moreover, its frequent downregulation in CRC suggests KCTD9 as a potential prognostic and therapeutic target in CRC.

## Introduction

According to the recent GLOBOCAN, 2020 estimates based on data from the International Agency for Research on Cancer (IARC), colorectal cancer (CRC) ranks third among all cancers in morbidity and second in mortality [1]. A large proportion of patients with CRC are diagnosed at advanced stages with mortality mainly associated with the high frequency of metastasis, particularly to the liver, in part because of direct circulation from the portal vein [2]. Early screening and improved management programs have helped to significantly improve patient prognosis [3], but there still remains an unmet need for patients with progressive disease. Consequently, a better understanding of the molecular mechanisms underlying CRC development and progression is needed to establish more effective biomarkers and treatment targets.

The Wnt signaling pathway is frequently activated in malignant cells and associated with the growth and metastasis of various tumor types including colon [4], liver [5], breast [6], stomach [7], salivary gland [8], and lung cancers [9]. Moreover, a recent review reflecting on decades of research denoted evidence for Wnt pathway involvement in all ten cancer hallmarks defined by Hanahan and Weinberg [10, 11]. Foremost, the Wnt pathway is involved in cell growth and differentiation, with the archetypal growth factor Wnt involved in directing organismal development through the control of tissue polarity via canonical and non-canonical (β-catenin-independent) mechanisms [12, 13]. Canonical Wnt signal transduction involves the cytoplasmic-to-nuclear translocation of β-catenin which then transactivates T cell factor/lymphoid enhancer factor (TCF/LEF)-dependent genes [14]. The stability of a β-catenin protein is crucial and mainly controlled by the APC/Axin (adenomatous polyposis coli) destruction complex wherein in the absence of Wnt, β-catenin interacts with the APC/Axin complex and undergoes phosphorylation, allowing its ubiquitination and proteasomal degradation [15, 16]. Notably, early studies showed that truncating mutations of the *APC* gene are highly prevalent in CRC and serve to stabilize β-catenin [17] and likewise stabilizing mutations of the β-catenin gene (*CTNNB1*) also occur but are not common in CRC [18]. However, these mutations cannot fully explain Wnt activation in CRC and the identification of other mechanisms has important implications for therapeutic interventions to target Wnt.

Of interest to this report is KCTD9, one of 25 members of the potassium channel tetramerization domain-containing (KCTD) family proteins, so named because they that share sequence similarities in their N-terminal domains with voltage-gated potassium channels [19]. KCTDs are intracellular proteins broadly distributed in the cytoplasm, mitochondria, and nucleus and play varying roles ranging from serving as auxiliary receptor subunits in GABA (γ-aminobutyric acid) signaling [20], cytoskeletal regulation [21], transcriptional regulation, and interaction with the cullin ubiquitin ligases to regulate protein ubiquitination and degradation [22]. Moreover, specific KCTD proteins have been implicated in a range of diseases, for example, the involvement of KCTD3 and KCTD7 in neurological disorders [19, 23, 24]. Many family members are also implicated in various cancers, either as tumor suppressors, oncogenes, or even both [25]. For instance, cooperation between KCTD15, KCTD6, KCTD11, and KCTD21 suppresses oncogenic signaling through the hedgehog pathway [26–28] whereas KCTD5 has been proposed to exhibit both tumor suppressive and oncogenic activity [25]. KCTD9 is primarily known for its association with hepatitis B virus-induced liver failure where KCTD9 is thought to play a causal role in NK cell activation [29]. However, the relevance of KCTD9 to tumor biology presently remains undefined although it would be expected to contribute some role given the importance of NK cells to antitumor immunity.

Here, using a combinatorial screen involving transcriptomic and patient survival data we identified KCTD9 as a potential biomarker of CRC. We found KCTD9 was commonly downregulated at the mRNA and protein level in CRC tissues and stratification according to KCTD9 expression showed that downregulation of KCTD9 was correlated with poorer patient outcomes. Functional investigations revealed that KCTD9 inhibits the proliferative and metastatic capacity of CRC cells both in vitro and in vivo. KCTD9 was shown to restrain the induction of Wnt signaling in CRC cells through control over the expression and activity of β-catenin. Mechanistically, this occurred via competition with β-catenin for binding to the zinc transporter protein ZNT9. Blocking this interaction renders β-catenin susceptible to proteasomal degradation with ensuant effects downstream to prevent the expression of Wnt pathway target genes. Together these findings identify KCTD9 as a novel tumor suppressor gene in CRC with practical implications for prognostic or therapeutic purposes.

## Results

### KCTD9 is commonly downregulated in colon adenocarcinoma tissues and cell lines

**Fig. 1:**
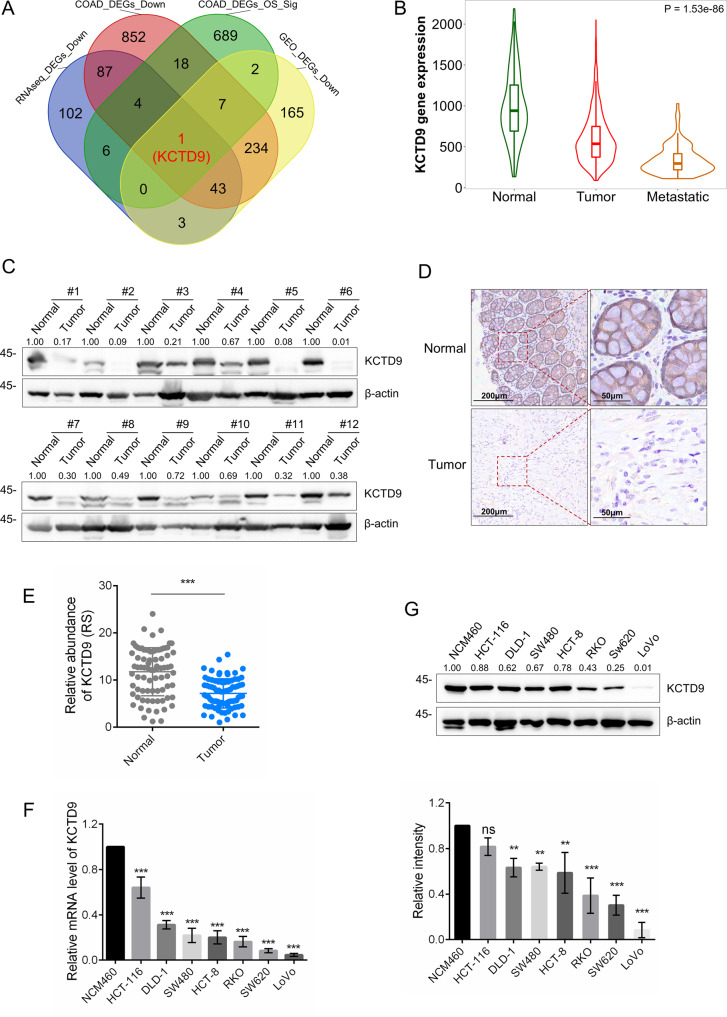
KCTD9 is downregulated in CRC. **A** Venn diagram showing intersecting downregulated differentially expressed genes (DEGs), as assessed by RNA sequencing (RNA-seq) results of three tissue samples from our hospital, and other public datasets: The Cancer Genome Atlas (TCGA)-COAD, GSE39582 and GSE75970. RNAseq_DEGs_Down: DEGs with consistently descending expressed trends among the adjacent normal colonic mucosa, primary colon adenocarcinoma and distant liver metastasis were identified from the RNA-seq data of three tissue samples in our hospital. COAD_DEGs_Down: DEGs downregulated between COAD *vs*. Normal groups based on TCGA-COAD data. COAD_DEGs_OS_Sig: DEGs significantly associated with overall survival (OS) of TCGA-COAD data predicted by both univariable cox and log-rank methods. GEO_DEGs_Down: Intersected DEGs downregulated between COAD/CRC *vs*. Normal groups based on GSE39582 and GSE75970 data. **B** Comparative expression of KCTD9 in normal colonic epithelium, primary and metastatic CRC tissues determined using the TNMplot web tool. **C** Western blot analysis of KCTD9 expression in 12 matched pairs of human CRC tissues and adjacent normal colonic tissues collected during resection (*n* = 12). β-actin was used as a loading control throughout. Relative density were quantified by using ImageJ software. **D**, **E** Tissue microarrays containing 80 pairs of human CRC tissues and adjacent normal colon tissues were analyzed for KCTD9 expression using immunohistochemical (IHC) staining. Representative staining in normal colon gland epithelium and adenocarcinoma tissues (**D**) (*n* = 80). Quantification of IHC staining in **D** as immunoreactive scores (IRS) **E** mean ± SD, *n* = 80, two-tailed Student’s *t* test, ***p < 0.001. Comparative analysis of KCTD9 expression in the normal human colon mucosal epithelial cell line NCM460 and a panel of CRC-derived cell lines using qRT-PCR (**F**) and Western blotting (**G**). **G** Is represent three independent experiments (top) and relative density were quantified by using ImageJ software (bottom). **F**, **G** Are mean ± SD; *n* = 3 independent experiments, one-way ANOVA with Tukey’s multiple comparison post-test, ns not significant, **p < 0.01, ***p < 0.001.

To identify genes that are potentially involved in CRC progression, we first sought to identify differentially expressed genes (DEGs) between adjacent normal colonic mucosa, primary colon adenocarcinoma, and distant liver metastasis (two groups were compared in each analysis, Supplementary Table 1). A total of 246 protein-coding DEGs with consistently decreasing expression trends in three samples were identified (threshold: padj <0.05 and log2 FoldChange < −1, Supplementary Table 2). In addition, 455 and 1246 downregulated DEGs (CRC *vs*. normal samples) were predicted by bioinformatics analysis of the GEO (GSE39582 and GSE75970 intersected) and TCGA-COAD datasets, respectively (Supplementary Table 3). Moreover, we used both univariable Cox and log-rank methods to predict 727 DEGs that were significantly associated with overall survival (OS) in the TCGA-COAD dataset (Supplementary Table 4). Finally, intersection analysis between the aforementioned screens provided a single common downregulated DEG, namely KCTD9 (Fig. 1A). On this basis, we focused our initial investigations on characterizing the expression of KCTD9 during CRC pathogenesis.

As expected, plots of the expression levels of KCTD9 mRNA in the GSE39582 and TCGA-COAD datasets showed that KCTD9 was significantly downregulated in CRC compared to normal tissues (*P* < 0.05 for all, Supplementary Fig. 1A, B). Moreover, independent microarray-based analyses verified that KCTD9 mRNA expression was progressively decreased in the transition of primary to metastatic cancer (Fig. 1B, Supplementary Table 5). It was then important to ascertain that the decreased KCTD9 transcript levels resulted in decreased protein levels. Towards this, we used Western blotting and immunohistochemistry to analyze the expression of KCTD9 in CRC versus normal adjacent tissues. Indeed, we observed a clear pattern where KCTD9 protein was reduced in 12 pairs of normal adjacent and CRC tissues (Fig. 1C) with a larger cohort of 80 patient cases revealing a similar reduction in KCTD9 immunostaining (Fig. 1D, E). Notably, the same findings were evident in cell culture models where the normal human colon mucosal epithelial cell line NCM460 expressed higher levels of KCTD9 mRNA and protein in comparison to CRC-derived cell lines (Fig. 1F, G). Taken together these data establish that KCTD9 is commonly downregulated in CRC.

### Downregulation of KCTD9 expression is correlated with disease progression and prognosis

**Fig. 2:**
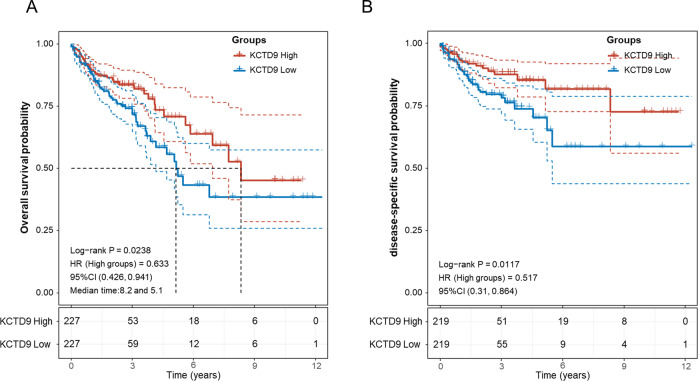
Downregulation of KCTD9 expression is correlated with disease progression and prognosis. **A**, **B** Kaplan-Meier analysis and log-rank test for CRC patients from the TCGA-COAD dataset stratified according to median KCTD9 expression levels. Cases with low KCTD9 expression were associated with significantly poorer overall survival (OS) (**A**) and disease-specific survival (DSS) (**B**).

We next dissected the clinical characteristics associated with KCTD9 expression available from the TCGA-COAD dataset. As anticipated, KCTD9 expression levels were reduced in tumors compared to normal tissues although the reductions were greater in patients showing disease progression (Supplementary Fig. 2A). Moreover, while the levels of KCTD9 were significantly reduced in both mucinous and adenocarcinoma subtypes, KCTD9 was more highly reduced in adenocarcinomas (Supplementary Fig. 2B). Decreasing trend of KCTD9 levels was mainly observed according to individual assessment of T, N, and M stages (Supplementary Fig. 2C–E). Furthermore, analysis of overall TNM staging showed there was a progressive downregulation of KCTD9 from stage I to stage IV cases (Supplementary Fig. 2F). Together these data suggest that KCTD9 downregulation occurs as an early transformation event but with levels continuing to diminish with disease progression. In addition, CRC patients presenting with high KCTD9 expression in primary tumors showed significantly better outcomes in terms of OS and DSS (Fig. 2A, B), indicating the contribution of KCTD9 loss to patient outcomes.

### KCTD9 inhibits CRC cell proliferation and metastasis in vitro

**Fig. 3:**
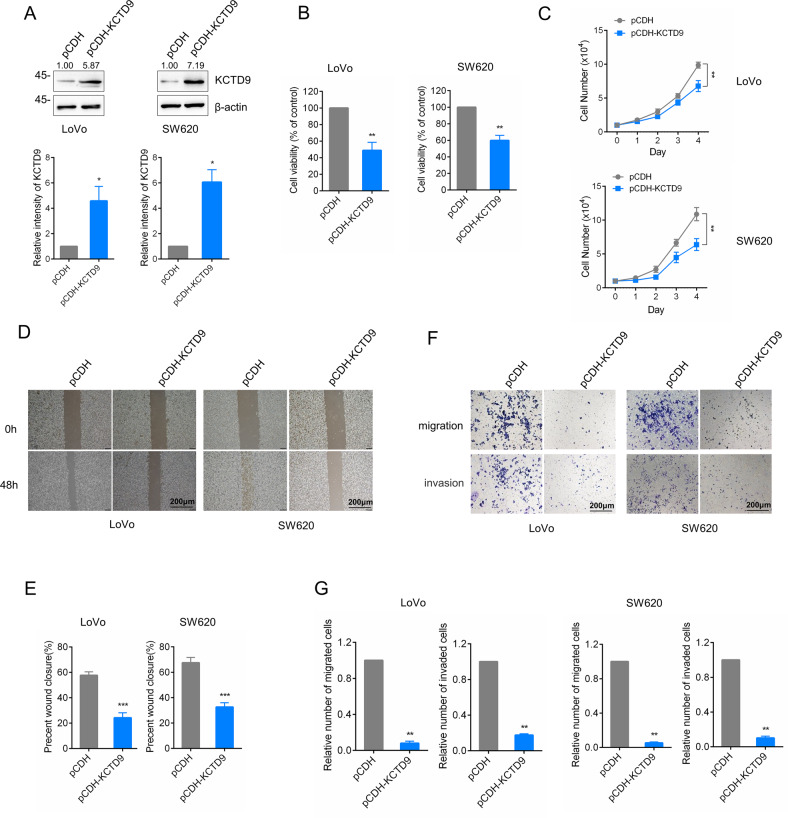
KCTD9 inhibits CRC cell proliferation and metastasis in vitro. **A** Western blotting analysis of the CRC cell lines LoVo and SW620 after infection with lentiviral particles based on control pCDH or pCDH-KCTD9 vectors to overexpress KCTD9. β-actin was used as a loading control. Data shown represent three independent experiments (top) and relative density were quantified by using ImageJ software (bottom). **B** Assessment of cell viability in the cells from **A** measured using CCK-8 assays. **C** Assessment of proliferation in the cells from **A** measured using Count Star (Countstar BioTech) over 1–4 days by cell counting. **D**, **E** Assessment of migration in the cells from **A** measured in wound healing assays. Representative images at time 0 and 48 h (scale bar=200 µm) (**D**) with quantitation of wound closure (**E**). **D** represent three independent experiments. **F**, **G** Assessment of migration and invasion in the cells from **A** using Transwell™ assays. Representative images of migrating/invading cells after 24 h (scale bar =200 µm) (**F**) with comparative quantitation of total migrating cells (**G**). **F** Represent three independent experiments. **A**, **B**, **E**, **G** Are mean ± SD, *n* = 3 independent experiments, two-tailed Student’s *t* test, *p < 0.05, **p < 0.01, ***p < 0.001. **C** is mean ± SD, *n* = 3 independent experiments, two-way ANOVA with Bonferroni’s multiple comparison post-test, **p < 0.01.

Based on the preceding data, we then sought to explore the role of KCTD9 in CRC progression using cell-based functional assays to measure malignant characteristics. Based on their low expression of KCTD9, we first overexpressed KCTD9 in the LoVo and SW620 CRC cell lines (Fig. 3A) and found that ectopic KCTD9 significantly decreased their cell viability and proliferative capacity (Fig. 3B, C). Similarly, assessment of cell motility and invasion in wound-healing and Transwell™ assays revealed that KCTD9 overexpression inhibited the metastatic potential of CRC cells (Fig. 3D–G). To then verify these effects, we used shRNA to knockdown KCTD9 in the HCT-116 and DLD-1 CRC cell lines that exhibited relatively high KCTD9 expression (Supplementary Fig. 3A). In contrast to the overexpression experiments, we found that KCTD9 knockdown accelerated cell proliferation (Supplementary Fig. 3B, C) and increased the rates of cell migration and invasion rates (Supplementary Fig. 3D–G). Notably, the use of two independent targeting sequences in these assays minimized the chances of off-target effects. Taken together, these findings indicate that KCTD9 represents a previously undiscovered tumor suppressor in CRC, acting to negatively regulate cell proliferation and metastasis.

### KCTD9 inhibits the EMT and Wnt signaling in CRC cells

**Fig. 4:**
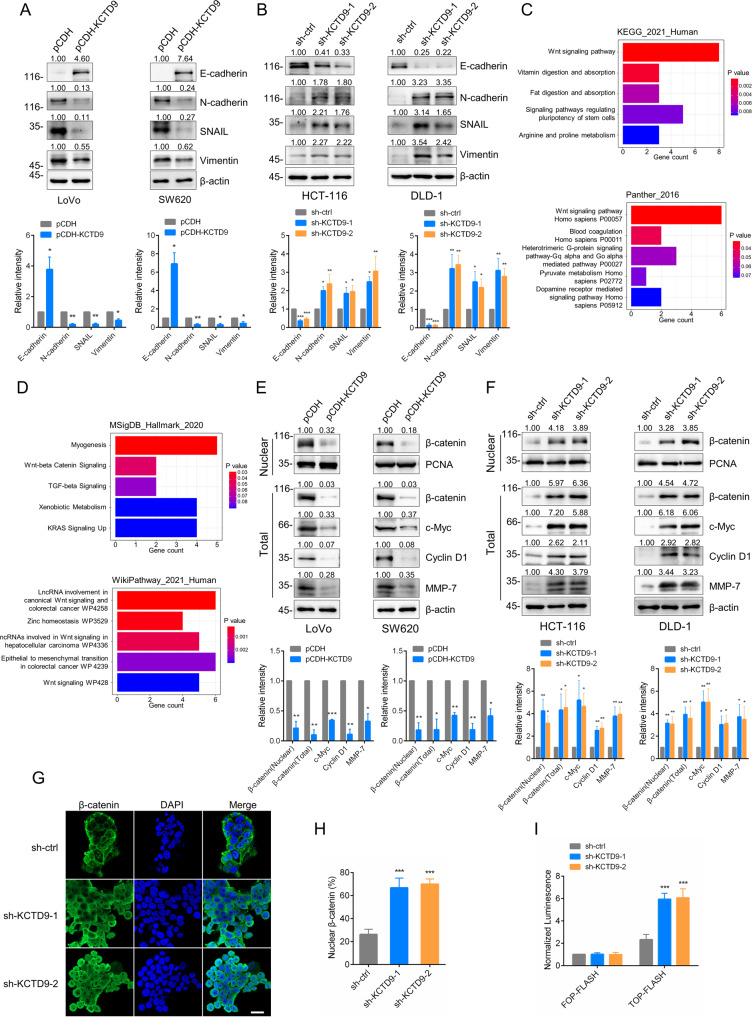
KCTD9 inhibits the EMT and Wnt signaling in CRC cells. **A**, **B** Western blotting analysis of the key EMT markers E-cadherin, N-cadherin, vimentin, and SNAIL in LoVo and SW620 cells after overexpression of KCTD9 or after knockdown of KCTD9 in HCT-116 and DLD-1 cells. β-actin was used as a loading control throughout. Data shown represent three independent experiments (top) and relative density were quantified by using ImageJ software (bottom). **C**, **D** Pathway enrichment analysis using the enrichR package. The TCGA-COAD dataset was reanalyzed based on low versus high KCTD9 expression and the 169 DEGs that were upregulated in low KCTD9 expressing CRC tumors used to interrogate four pathway enrichment annotation databases (KEGG_2021_Human, Panther_2016, MSigDB_Hallmark_2020, and WikiPathway_2021_Human). **E**, **F** Western blotting analysis of β-catenin, c-Myc, cyclin D1, and MMP-7 in total cell lysates and β-catenin in nuclear isolates after overexpression of KCTD9 in LoVo and SW620, or after knockdown of KCTD9 in HCT-116 and DLD-1 cells . PCNA was used as a loading control for nuclear proteins. Data shown represent three independent experiments (top) and relative density were quantified by using ImageJ software (bottom). **G**, **H** Control or KCTD9-knockdown HCT-116 cells were subjected to immunofluorescence staining against β-catenin. Representative confocal images show β-catenin staining alone (green) or in combination with nuclear staining using DAPI (blue) (**G**). The percentage of cells with coincident staining of β-catenin in the nucleus was quantitated (**H**). **G** Represent three independent experiments. **I** TOP Flash/FOP Flash luciferase reporter assays were conducted in the indicated control or KCTD9-knockdown HCT-116 cells to measure TCF/LEF transcriptional activity. **A**, **E** are mean ± SD, *n* = 3 independent experiments, two-tailed Student’s *t* test, ^*^p < 0.05, **p < 0.01, ***p < 0.001. **B**, **F**, **H** are mean ± SD; *n* = 3 independent experiments, one-way ANOVA with Tukey’s multiple comparison post-test, ^*^p < 0.05, **p < 0.01, ***p < 0.001. **I** Is mean ± SD, *n* = 3 independent experiments, two-way ANOVA with Bonferroni’s multiple comparison post-test, ***p < 0.001.

One of the well-known drivers of CRC progression is the epithelial-mesenchymal transition (EMT) [30]. We hypothesized that the EMT could represent the underlying mechanism whereby KCTD9 loss impacts the phenotypic changes in CRC cells. Indeed, examination of the expression of key EMT markers by Western blotting showed changes consistent with EMT regulation. On the one hand, overexpression of KCTD9 enhanced the expression of the epithelial marker E-cadherin and reduced the expression of N-cadherin, SNAIL, and vimentin in LoVo and SW620 cells (Fig. 4A) whereas silencing of KCTD9 in HCT-116 and DLD-1 cells showed the opposite effects with decreases in E-cadherin and increases in N-cadherin, SNAIL, and vimentin (Fig. 4B). Therefore, we concluded that KCTD9 inhibits the EMT process in CRC cells.

To further dissect the regulatory pathways involved, we reanalyzed the TCGA-COAD dataset based on low versus high expression of KCTD9. This produced a total of 169 DEGs (FoldChange > 1.5 & adj. *P* Value < 0.05) that were upregulated in low KCTD9 expressing tumors (Supplementary Table 6). We then employed the enrichR package to survey the impact of the upregulated DEGs on different signaling pathways. The results revealed significant associations with Wnt signaling among four different pathway enrichment databases along with the ‘EMT in CRC’ annotation from the WikiPathway (Fig. 4C, D). Further examination of Wnt pathway activation components including c-Myc, cyclin D1, and MMP-7 showed these markers all decreased when KCTD9 was overexpressed in LoVo and SW620 cells (Fig. 4E). And conversely, all markers increased after the knockdown of KCTD9 in HCT-116 and DLD-1 cells (Fig. 4F). Moreover, changes in these markers were mirrored by β-catenin, the key mediator of Wnt signaling, and instructively, subcellular fractionation assays showed that the levels of nuclear β-catenin were negatively modulated by KCTD9 expression (Fig. 4E, F). Similarly, immunofluorescence analysis showed that the proportion of β-catenin in the nucleus increased after KCTD9 knockdown in HCT-116 cells (Fig. 4G, H). Lastly, confirmation of transcriptional activity changes in Wnt signaling target genes was confirmed in TOP/FOP Flash reporter assays conducted in HCT-116 cells where luciferase activity was increased threefold upon KCTD9 knockdown (Fig. 4I). Consistent with the actions of KCTD9 influencing transcriptional activity, it was observed that the protein expression changes in c-Myc, cyclin D1, and MMP-7 were reflected in alterations at the transcriptional level (Supplementary Fig. 4A–D).

Collectively these data propose that KCTD9 inhibits the EMT in CRC cells by inhibition of Wnt signaling with loss of KCTD9 resulting in the redistribution of β-catenin to the nucleus with ensuing Wnt activation. But how KCTD9 functions in this context, however, remained to be determined.

### KCTD9 binding to ZNT9 competes with ZNT9 binding to β-catenin

**Fig. 5:**
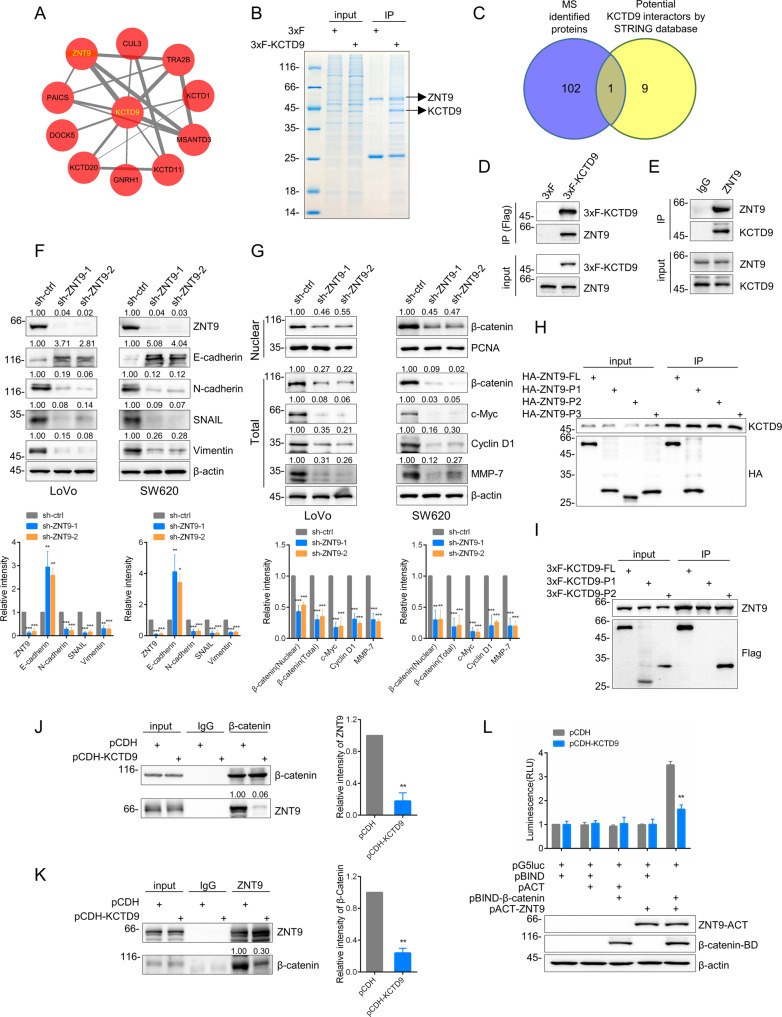
KCTD9 binding to ZNT9 competes with ZNT9 binding to β-catenin. **A** Protein-protein interaction network predicted for KCTD9 using the Search Tool for Retrieval of Interacting Genes/Proteins (STRING) database. **B** Recombinant Flag-tagged (3xF)-KCTD9 was immunoprecipitated from HCT-116 cells to identify putative KCTD9-binding proteins. Coomassie blue-stained SDS-PAGE gel compares control IgG versus Flag immunoprecipitates with the highlighted bands identified as KCTD9 and ZNT9 using mass spectrometry. Data shown represent three independent experiments. **C** Venn diagram showing intersecting candidate KCTD9-binding proteins identified from the analyses in **A**, **B**. **D**, **E** Western blotting verification that ZNT9 co-precipitates with ectopically expressed 3xF-KCTD9 and conversely that KCTD9 co-precipitates with ZNT9 in HCT-116 cells. Data shown represent three independent experiments. **F** Western blotting analysis of ZNT9, E-cadherin, N-cadherin, vimentin, and SNAIL in LoVo and SW620 cells after knockdown of ZNT9. Data shown represent three independent experiments (top) and relative density were quantified by using ImageJ software (bottom). **G** Western blotting analysis of β-catenin, c-Myc, cyclin D1, and MMP-7 in total cell lysates and β-catenin in nuclear isolates of the ZNT9 knockdown cells from (**F**). β-actin and PCNA were used as loading controls for total and nuclear proteins, respectively. Data shown represent three independent experiments (top) and relative density were quantified by using ImageJ software (bottom). **H**, **I** SW620 cells were transfected with the indicated constructs and immunoprecipitation (IP) analyses used to detect protein-domain interactions between KCTD9 and the indicated HA-tagged constructs of ZNT9 (FL, P1, P2, and P3) or ZNT9 and the indicated Flag-tagged constructs of KCTD9 (FL, P1, and P2). Data shown represent three independent experiments. **J**, **K** Co-immunoprecipitation assays were conducted in SW620 cells measuring interactions between β-catenin and ZNT9 and ZNT9 and β-catenin in the absence or presence of KCTD9 overexpression. Data shown represent three independent experiments (left) and relative density were quantified by using ImageJ software (right). **L** Mammalian two-hybrid assays in SW620 cells measuring interactions between ZNT9 and β-catenin in the absence or presence of KCTD9 overexpression. **F**, **G** are mean ± SD; *n* = 3 independent experiments, one-way ANOVA with Tukey’s multiple comparison post-test, *p < 0.05, **p < 0.01, ***p < 0.001. **J**, **K** are mean ± SD, *n* = 3 independent experiments, two-tailed Student’s *t* test, **p < 0.01. **L** Is mean ± SD, *n* = 3 independent experiments, two-way ANOVA with Bonferroni’s multiple comparison post-test, **p < 0.01.

We considered that identifying the protein interaction partners of KCTD9 would likely be important in understanding how KCTD9 influences Wnt signaling. Bioinformatics analysis using the STRING database provided a shortlist of 10 candidate genes (Fig. 5A, Supplementary Table 7) while mass spectrometry-based experiments to identify KCTD9-interacting proteins in HCT-116 cells uncovered 103 candidate proteins (Fig. 5B, Supplementary Table 8). The intersection between these lists provided only a single candidate in common (Fig. 5C), ZNT9 a zinc transporter protein involved in intracellular zinc homeostasis [31]. Our interest was further piqued given that ZNT9 had been previously implicated in the regulation of TCF/LEF target gene expression as a binding partner of β-catenin [32]. Follow-up immunoprecipitation experiments then confirmed that ectopically expressed flag-KCTD9 robustly recovered ZNT9 from HCT-116 cells (Fig. 5D) while conversely endogenous KCTD9 was recovered in ZNT9 immunoprecipitates (Fig. 5E). Thus, we confirmed that ZNT9 was a bona fide interacting partner of KCTD9 in CRC cells.

To next validate that ZNT9 was involved in the EMT via Wnt signaling in CRC cells, we examined the expression of key EMT and Wnt pathway activation markers after manipulating ZNT9 expression. Notably, knockdown of ZNT9 in LoVo and SW620 cells resulted in increased protein expression of E-cadherin but conversely decreased expression of N-cadherin, SNAIL, and vimentin (Fig. 5F). Moreover, knockdown of ZNT9 was associated with decreases in the protein expression of c-Myc, cyclin D1, and MMP-7 along with decreases in the levels of total and nuclear β-catenin (Fig. 5G). Furthermore, there were consistent decreases in the mRNA levels of c-Myc, cyclin D1, and MMP-7 (Supplementary Fig. 5A, B), indicating that ZNT9 functions as a positive regulator of Wnt/β-catenin signaling and the EMT in CRC cells.

We then returned to consider the association between KCTD9 and ZNT9, using mapping experiments to better define the nature of their interaction. Towards this, we constructed domain truncation mutants of KCTD9 and ZNT9 using Flag and HA epitope tags to delineate each protein, respectively (Supplementary Fig. 5C, D). The construction design of KCTD9 was based on two domains, the N-terminal BTB domain and variable C-terminal sequence [33] while ZNT9 was divided into three fragments involving its N-terminal region (amino acids 1-200), central region (amino acids 201-370) and C-terminal region (amino acids 371-567). Co-transfection experiments with these constructs showed that the N-terminal domain of ZNT9 interacted with KCTD9 (Fig. 5H) while the C-terminal region of KCTD9 interacted with ZNT9 (Fig. 5I). As prior studies had shown that β-catenin binds directly to the N-terminus of ZNT9 [33] we speculated that KCTD9 competes with β-catenin for binding to ZNT9, resulting in inhibition of Wnt/β-catenin signaling.

To examine this possibility, we undertook co-immunoprecipitation analyses in CRC cells without and with ectopic expression of KCTD9. Indeed, it was observed that overexpression of KCTD9 markedly reduced the recovery of ZNT9 in β-catenin immunoprecipitates (Fig. 5J) and similarly reduced the amount of β-catenin recovered with ZNT9 (Fig. 5K). This result was independently substantiated using mammalian 2-hybrid assays where the overexpression of KCTD9 significantly decreased the interaction levels between ZNT9 and β-catenin (Fig. 5L). Together these results provide strong evidence that KCTD9 functions as a negative regulator of Wnt/β-catenin signaling by competing with β-catenin binding to ZNT9.

### KCTD9 regulates the stability of β-catenin

**Fig. 6:**
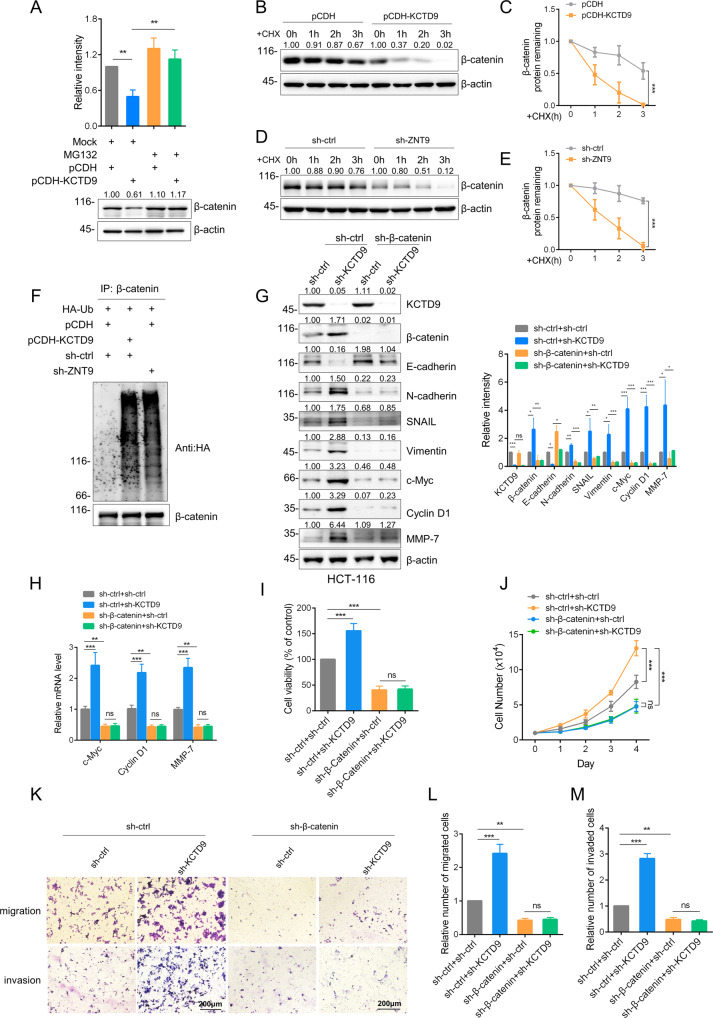
KCTD9 regulates the stability of β-catenin. **A** Western blot analysis comparing the expression of β-catenin in SW620 cells without (pCDH) or with overexpression of KCTD9 (pCDH-KCTD9) in untreated or MG132 treated cells (10 μM MG132 for 12 h). β-actin was used as a loading control throughout. Data shown represent three independent experiments (bottom) and relative density were quantified by using ImageJ software (top). **B**, **C** Western blot comparing the expression of β-catenin in SW620 cells without (pCDH) or with overexpression of KCTD9 (pCDH-KCTD9) after treatment with cycloheximide (50 μg/ml) for the indicated times. Accompanying densitometric quantitation of β-catenin relative to β-actin (**C**). **D**, **E** Western blot comparing the expression of β-catenin in SW620 cells without (sh-ctrl) or with knockdown of ZNT9 (shZNT9) after treatment with cycloheximide (50 μg/ml) for the indicated times. Accompanying densitometric quantitation of β-catenin relative to β-actin (**E**). **D** Represent three independent experiments. **F** SW620 cells were infected with the indicated combinations of sh-ctrl, sh-ZNT9, pCDH, or pCDH-KCTD9 lentiviruses, followed by co-transfection with HA-ubiquitin. Cells were then pretreated with MG132 (10 μM) for 12 h before immunoprecipitating β-catenin with polyubiquitination detected using anti-HA antibodies. Data shown represent three independent experiments. **G** Western blot comparing the levels of KCTD9, β-catenin, E-cadherin, N-Cadherin, SNAIL, vimentin, c-Myc, cyclin D1, and MMP-7 in HCT-116 cells infected with the indicated combinations of sh-ctrl, sh-KCTD9, and sh-β-catenin lentiviruses. Data shown represent three independent experiments (left) and relative density were quantified by using ImageJ software (right). **H** qPCR analysis of samples from **G** comparing the mRNA levels of c-Myc, cyclin D1, and MMP-7. **I** Assessment of cell viability in the cells from **G** measured using CCK-8 assays. **J** Assessment of proliferation in the cells from **G** measured using Count Star (Countstar BioTech) over 1–4 days by cell counting. **K**–**M** Assessment of migration and invasion in the cells from **G** using Transwell™ assays. Representative images of migrating/invading cells after 24 h (scale bar=200 µm) **K** with comparative quantitation of total migrating (**L**) or invading (**M**) cells. **K** Represent three independent experiments. **A**, **G**, **I**, **L**, **M** are mean ± SD; *n* = 3 independent experiments, one-way ANOVA with Tukey’s multiple comparison post-test, ns, not significant, ^*^p < 0.05, **p < 0.01, ***p < 0.001. **C**, **E**, **H**, **J** are mean ± SD, *n* = 3 independent experiments, two-way ANOVA with Bonferroni’s multiple comparison post-test, ns not significant, **p < 0.01, ***p < 0.001.

From the preceding experiments, it was evident that ZNT9 and KCTD9 both impacted the nuclear localization of β-catenin but both proteins also affected total β-catenin levels, positively and negatively, respectively. This suggested that β-catenin was stabilized by ZNT9 with competitive binding by KCTD9 rendering β-catenin susceptible to degradation. Supporting this notion, treating SW620 cells with the proteasomal inhibitor MG132 served to stabilize the reductions in β-catenin levels following the KCTD9 overexpression (Fig. 6A). Consistently, the half-life of β-catenin was drastically reduced in KCTD9-overexpressing cells (Fig. 6B, C). Similarly, the half-life of β-catenin was compromised when ZNT9 was knocked down in SW620 cells (Fig. 6D, E). Confirming these effects were dependent upon proteasomal-mediated degradation, the polyubiquitination of β-catenin was markedly increased when either KCTD9 was overexpressed or when ZNT9 was knocked down in SW620 cells (Fig. 6F). Moreover, there appeared to be no feedback evidence between KCTD9 and β-catenin since the knockdown of the latter did not influence KCTD9 levels (Supplementary Fig. 6A). Taken together with the preceding results, these data establish that the KCTD9-ZNT9 interaction fundamentally acts to destabilize β-catenin levels in CRC cells.

To next reconcile the evidence that Wnt signaling regulation by KCTD9 involves β-catenin, we examined hierarchical dependencies in downstream gene regulation using co-transfection experiments. First, we found that changes associated with the knockdown of KCTD9, namely the decreased levels of E-cadherin and increased N-cadherin, SNAIL, vimentin, c-Myc, cyclin D1, and MMP-7, were all nullified when β-catenin was silenced in HCT-116 cells (Fig. 6G). Moreover, the changes in c-Myc, cyclin D1, and MMP-7 protein levels were reflected in alterations in mRNA abundance (Fig. 6H), indicative that transcriptional regulation underlies these changes. Furthermore, knockdown of KCTD9 was similarly unable to reverse the ZNT9-knockdown dependent changes in these markers, either at the protein or transcript levels (Supplementary Fig. 6B, C). This established that the gene regulatory effects of KCTD9 and ZNT9 were dependent on β-catenin transcriptional activity.

In parallel with these experiments, we also determined whether the gene expression changes observed matched with the findings of functional assays measuring CRC cell proliferation and motility. Knockdown of β-catenin completely ablated the increased cell proliferation, migration, and invasion resulting from KCTD9 knockdown in HCT-116 cells (Fig. 6J–M), and similarly, KCTD9 knockdown effects were overridden by knockdown of ZNT9 (Supplementary Fig. 6E, F, D–H). Additionally, we checked the β-catenin and ZNT9 expression levels in normal human colon mucosal epithelial cell line and CRC-derived cell lines. We found that β-catenin expression was negatively correlated with KCTD9 expression, while ZNT9 levels were shown no correlation with β-catenin or KCTD9 expression (Supplementary Fig. 6I). Thus, β-catenin represents the key target associated with the tumor-suppressive activity of KCTD9.

### KCTD9 inhibits CRC growth and metastasis in vivo

**Fig. 7:**
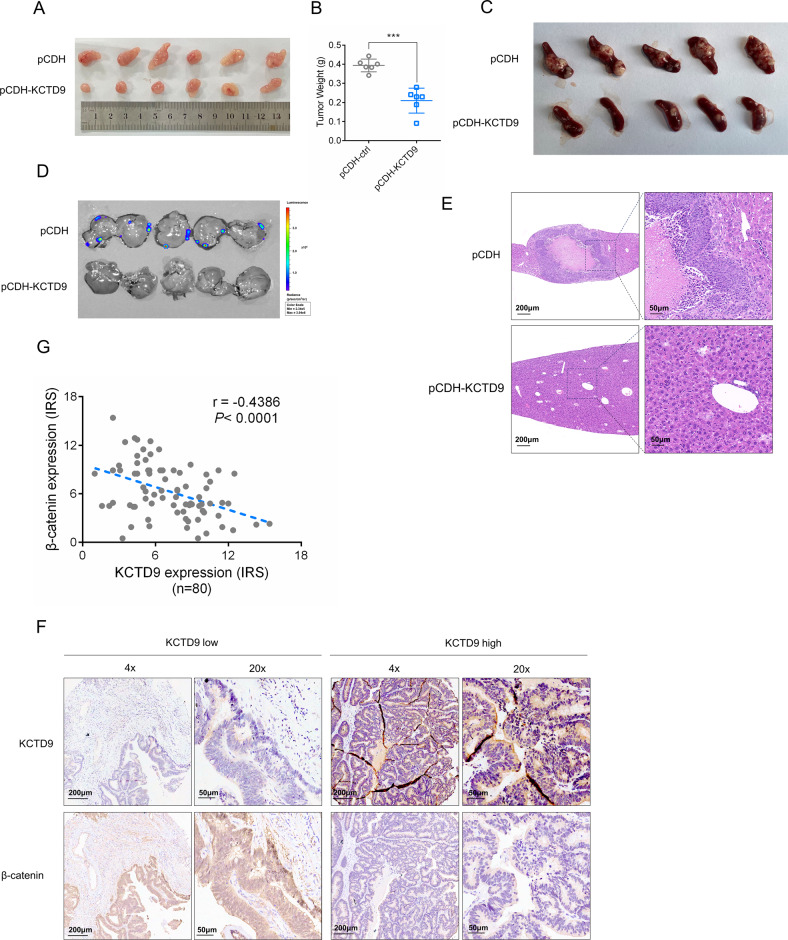
KCTD9 inhibits CRC growth and metastasis in vivo. **A**, **B** SW620 cells without (pCDH) or with overexpression KCTD9 (pCDH-KCTD9) were subcutaneously inoculated into nude mice. Images (**A**) and weights (**B**) of the excised xenografts recovered at 21 days. Data represent mean ± SD, *n* = 6 mice per group, two-tailed Student’s *t* test, ***p < 0.001. **C**–**E** SW620 cells without (pCDH) or with overexpression KCTD9 (pCDH-KCTD9) were inoculated under the spleen capsule of nude mice to measure spleen to liver metastasis using a luciferase-based bioluminescence assay. Images of the excised spleens recovered at 28 days (**C**) along with bioluminescence images of whole livers (**D**) and hematoxylin and eosin (**E**) stained sections of liver tissue. *n* = 5 mice per group. **F**, **G** Tissue microarrays containing 80 human CRC tissues were analyzed in parallel for expression of KCTD9 and β-catenin using immunohistochemical (IHC) staining. Representative staining of cases with low and high expression of KCTD9, respectively (**F**) and quantification of IHC staining as immunoreactive scores (IRS) (**G**). The Pearson correlation coefficient (r) was calculated as shown. (*n* = 80).

To provide further functional evidence supporting the general importance of *KCTD9* as a tumor suppressor gene in CRC, we examined the growth and metastasis of SW620 cells in vivo with and without overexpression of KCTD9. Notably, the tumors established after KCTD9-overexpression were significantly smaller after subcutaneous implantation in nude mice (Fig. 7A, B). Furthermore, the same result was evident in the primary tumors formed in a spleen-liver metastasis model (Fig. 7C) where it was evident that metastasis to the liver was significantly decreased after overexpression of KCTD9 (Fig. 7D, E). Therefore, the tumor suppressor activity of KCTD9 in CRC cells can be readily reproduced in an in vivo setting.

Finally, we returned to consider the clinical relevance of the KCTD9-dependent regulation of β-catenin in human CRCs. If the mechanism was relevant in this setting it would be predicted that the expression of KCTD9 would be negatively correlated with that of β-catenin. Towards this notion, we used immunohistochemistry to measure the expression of KCTD9 in a primary CRC tissue array containing 80 case samples. This analysis showed a clear trend that CRC tissues with low KCTD9 expression had higher β-catenin expression while the converse was true for CRC tissues with high KCTD9 expression (Fig. 7F). Quantitative assessment of staining as IRS showed that KCTD9 was negatively correlated with β-catenin expression (Fig. 7G). Interestingly, we found that single or double mutations of APC and CTNNB1 which are known to affect the levels of β-catenin in CRC cells [17, 18] were not associated with the levels of KCTD9 (Supplementary Fig. 7A). Thus, it appears likely that the downregulation of KCTD9 occurs independently of such mutations and the effects of KCTD9 on β-catenin expression would still be relevant in APC and/or CTNNB1 mutant cancers. Collectively, these data provide substantive evidence as to the relevance of KCTD9 expression towards human CRC.

## Discussion

Wnt signaling is a fundamental signaling pathway required for correct development along with tissue homeostasis with important roles in stem cell renewal and organ regeneration. Activation of Wnt signaling is tightly regulated through the expression and cellular location of the transcription factor β-catenin with increased nuclear expression associated with Wnt signal transduction. It is not surprising that Wnt/β-catenin signaling is also important in tumorigenesis, and indeed when Wnt/β-catenin signaling is inhibited, cell proliferation and metastasis are suppressed in a variety of tumors [34–38]. On this basis, efforts to target the Wnt pathway as a cancer therapy are ongoing. For example, Toosendanin (TSN), a novel WW Domain Containing Oxidoreductase (WWOX) candidate agonist, has significant anti-proliferative and anti-metastatic effects on liver cancers, and functions by accelerating the degradation of β-catenin by promoting the activity of the degradation complex components including APC, AXIN1, CK1 and the GSK3β complex [34]. In this paper, we identified KCTD9 as a novel tumor suppressor in CRC that similarly acts to increase the degradation of β-catenin.

KCTD9 is the only human protein containing five pentapeptide repeats (presumably a DNA mimicking structure) [33] and has been described to play a role in NK cell lineage commitment and maturation. Notably, experiments in mice showed KCTD9 to mediate direct or indirect interaction-dependent degradation of transcription factors or chromatin regulators leading to marked increases in NK cell CD69 expression, cytotoxicity, IFN‐γ secretion with significant decreases in NKG2A receptor expression [39]. Moreover, KCTD9 was highly expressed in hepatic NK cells obtained from HBV-ACLF patients compared with that in mild chronic hepatitis B (CHB) patients, indicating KCTD9 is a potential therapeutic target in HBV-ACLF patients [29]. No information has presently been published regarding KCTD9 and cancer although one recent article projected that KCTD9 would likely function in an oncogenic role [25]. Given that NK cells are a key immune constituent in the protective antitumor immune response we would expect a role for KCTD9 in tumorigenesis [40]. Nevertheless, while our data show that KCTD9 does mediate the degradation of the transcription factor β-catenin, our results establish an entirely different role for KCTD9 in CRC cells as a tumor suppressor.

Critical to the actions of KCTD9 was its interaction with ZNT9, also known as SLC30A9 and GAC63. An early biochemical report showed that ZNT9 enhanced TCF/LEF-regulated gene transcription with ZNT9 binding to β-catenin shown to co-occupy the promoter of the Wnt target gene *cyclin D1* [33]. A more recent transcriptomic analysis of zinc transporters in CRC also concluded that ZNT9 expression was correlated with some Wnt pathway elements, lending support to the concept that ZNT9 functions as a positive regulator of Wnt signaling [41]. Our investigations were able to substantiate that ZNT9 does regulate the Wnt pathway in CRC while also providing the mechanistic basis of how this process is regulated. Specifically, we showed binding between KCTD9 and ZNT9 was able to disrupt the association between ZNT9 and β-catenin. Therefore, it is conceivable that when KCTD9 levels are low, β-catenin interacts with ZNT9 and then, in conjunction with TCF/LEF, activates the transcription of downstream genes, including *c-Myc, cyclin D1*, and *MMP-7*. However, when KCTD9 levels are high, KCTD9 competes with β-catenin for ZNT9 binding, resulting in the dissociation of the ZNT9-β-catenin complex and the subsequent inactivation of TCF/LEF-dependent gene transcription. Our working model for the KCTD9-dependent regulation of Wnt/β-catenin signaling is illustrated in Supplementary Fig. 7B.

Finally, we must consider the clinical significance of the findings of our study. Foremost, KCTD9 expression appears to be reduced after CRC transformation and further lowered during disease progression. Moreover, KCTD9 downregulation appears a rather common event in CRC and since its downregulation occurs at the mRNA and protein levels this suggests gene-level effects although this notion remains to be investigated. Irrespectively, we demonstrate this event has a significant impact on patients with an association with worsened clinical parameters and outcomes. Together this proposes KCTD9 as a potential prognostic and therapeutic target in CRC. Furthermore, based on our findings it would also be relevant to study the expression status of KCTD9 in other cancer types, particularly those which are known to be driven by aberrant Wnt signaling.

## Materials and methods

### RNA sequencing (RNA-seq)

Three tissue samples used in RNA sequencing, including adjacent normal colonic mucosa, primary colon adenocarcinoma and distant liver metastasis, were collected from one patient in our hospital who was diagnosed with stage IV colon adenocarcinoma. Total RNA was extracted from the above three samples with TRIzol according to the manufacturer’s protocol (Invitrogen). RNA degradation and contamination was monitored on 1% agarose gels. RNA purity was checked using the NanoPhotometer ^®^ spectrophotometer (IMPLEN, CA, USA). RNA integrity was assessed using the RNA Nano 6000 Assay Kit of the Bioanalyzer 2100 system (Agilent Technologies, CA, USA).

A total amount of 1 µg RNA per sample was used as input material for the RNA sample preparations. Sequencing libraries were generated using NEBNext^®^ UltraTM RNA Library Prep Kit for Illumina^®^ (NEB, USA) following the manufacturer’s recommendations and index codes were added to attribute sequences to each sample. Prior to differential gene expression analysis, for each sequenced library, the read counts were adjusted by edgeR program package through one scaling normalized factor. Two-by-two comparisons were made between the three samples to obtain the differential genes by using the edgeR R package (version 3.18.1). The *P* values were adjusted using the Benjamini & Hochberg method. Adjusted *P*-value of 0.05 and absolute log_2_FoldChange of 1 was set as the threshold for significantly differential expression. The RNA-seq data was submitted to GEO (Gene Expression Omnibus) database (Accession number: GSE205547). The clinical synopsis of the patients’ cohort used for RNA sequencing was shown in Supplementary Table 9.

### Public datasets collection and bioinformatics analysis

The TCGA-COAD, GSE39582 [42] and GSE75970 datasets were retrieved from the Genomic Data Commons Data Portal (https://portal.gdc.cancer.gov/) and Gene Expression Omnibus (GEO, https://www.ncbi.nlm.nih.gov/geo/), respectively. Differentially expressed genes (DEGs) between the CRC and normal groups for each dataset were identified by the limma R package (version 3.40.2) and GEO2R online tool (https://www.ncbi.nlm.nih.gov/geo/geo2r/). In addition, TNMplot (https://tnmplot.com/analysis/) [43], a web tool for differential gene expression analysis in tumors, normal and metastatic tissues, was used to validate KCTD9 differential expression. Correlations between *KCTD9* gene expression level and patients’ clinical characteristics in the TCGA-COAD dataset, including age, sex, tumor (T) stage, lymph node (N) stage, metastasis (M) stage, etc., were analyzed with the ggstatsplot R package [44]. Clinical data were downloaded from TCGA and the Pan-Cancer Atlas [45] on the UCSC Xena website (https://xenabrowser.net/) to analyze the prognostic value of differential *KCTD9* gene expression levels in the TCGA-COAD dataset concerning overall survival (OS) and disease-specific survival (DSS). The R packages survival and survminer were used to draw Kaplan-Meier survival curves.

Where indicated, pathway enrichment analysis was performed against specified DEG lists using the Enrichr database (https://maayanlab.cloud/Enrichr/) [46–48] and enrichR package. For protein-protein interaction (PPI) network analysis, the Search Tool for the Retrieval of Interacting Genes/Proteins (STRING) database (https://string-db.org) was used [49]. The file obtained in TSV format was visualized with Cytoscape software (version 3.8.2) [50].

### Cell lines and human tissues

All cell lines were cultured in the specified media containing 10% FBS and 1% penicillin/streptomycin at 37 °C in a 5% CO_2_ environment. HEK293T, NCM460, and HCT-116 cells were cultured in DMEM, SW480, and SW620 cells in L-15, DLD-1 in RPMI-1640, and LoVo cells in F-12K media. All cells were tested for mycoplasma contamination by RT-PCR. Alternatively, fresh human colon cancer and adjacent noncancerous tissues were obtained from patients undergoing surgical resection at the First Affiliated Hospital of the University of Science and Technology of China (USTC, Hefei, China). The experiments using fresh human tissues and human CRC tissue microarrays (Outdo Biotech, Shanghai, China) were approved by the Human Research Ethics Committees of the University of Science and Technology of China in agreement with the guidelines outlined in the Declaration of Helsinki. The clinical synopsis of the patients’ cohort used in the study was shown in Supplementary Table9.

### Immunohistochemistry

Human CRC tissue microarrays (obtained from Outdo Biotech, Shanghai, China) were stained with antibodies against KCTD9 (Abcam, 1:300, ab180937) or β-catenin (Proteintech, 1:1000, 51067-2-AP). Images were captured using an AxioVision Rel.4.6 computerized image analysis system (Carl Zeiss Co. Ltd., Jena, Germany) with immunoreactive scores (IRS) calculated as the product of the staining intensity score and the proportion of positively stained cells. Alternatively, hematoxylin and eosin (H&E) staining was performed. The clinical synopsis of the patients’ cohort used in the study was shown in Supplementary Table 9.

### Cell viability and cell proliferation assays

SW620, LOVO, HCT-116 and DLD-1 cells were seeded at a density of 3× 10^3^ cells per well in 96-well plates. Cell viability was determined by Counting Kit-8 (CCK-8; Beyotime, Shanghai, China) assay the day after seeding, according to the manufacturer’s protocols. In summary, 10 μL of CCK-8 solution was added to 90 μL of cell medium and incubated for 3 h in a 5% CO2 and 37 °C environment. Then, the absorbance was measured at 450 nm with a microplate reader (Bio-Tek, Winooski, VT, USA). Cells for proliferation assays were seeded at a density of 1× 10^4^ cells per well in 12-well plates and the cells were counted by using Count Star (Countstar BioTech) on day 1, day 2, day 3 and day 4 after trypsin digestion.

### Western blot and immunoprecipitation

Total cell lysates were obtained using RIPA buffer (Beyotime, Shanghai, China) supplemented with protease inhibitors. After heating at 95 °C, the supernatant samples were subjected to SDS-PAGE with proteins then transferred to nitrocellulose membranes. Thereafter, the membranes were blocked with 5% nonfat milk, incubated with primary and HRP-conjugated secondary antibodies, and reactive bands visualized with enhanced chemiluminescence (Tanon 5200). Antibodies used for Western blotting were against KCTD9 (Abcam, 1:1000, ab180937), ZNT9 (SANTA CRUZ, 1:1000, sc-271956), N-CAD (Proteintech, 1:1000, 22018-1-AP), E-CAD (Proteintech, 1:1000, 20874-1-AP), SNAIL (Proteintech, 1:1000, 13099-1-AP), vimentin (Proteintech, 1:1000, 10366-1-AP), c-Myc (Proteintech, 1:1000, 67447-1-Ig), β-catenin (Proteintech, 1:1000, 51067-2-AP), Flag (Abmart, 1:2500, M20026), HA (Abmart, 1:1000, M20021), cyclin D1 (Proteintech, 1:2000, 60186-1-Ig), MMP7 (Proteintech, 1:1000, 10374-2-AP), PCNA (Proteintech, 1:2000, 10205-2-AP), β-actin (Proteintech, 1:1000, 66009-1-Ig), Goat Anti-Mouse lgG HRP (light chain specific, Abmart 1:3000, M21004), Anti-Flag-Tag Mouse mAb (Agarose Conjugated, Abmart M20018) and Alexa Fluor 488-labeled Goat Anti-Rabbit IgG(H + L) (Beyotime, 1:500, A0423). Alternatively, immunoprecipitation samples were prepared from cells that were lysed in IP lysis buffer (0.5% NP-40, 0.5% Triton-X100, 1.5 mM MgCl_2_, 150 mM NaCl, and 20 mM Tris-HCl at pH 7.3, and 10% glycerin) containing a protease inhibitor cocktail for 35 min on ice. The cell lysates were incubated with 1~2 µg/ml antibody at 4 °C for 4 h and then recovered with protein A/G Sepharose beads for 2 h. After washing three times with IP lysis buffer and boiling at 95 °C for 10 min, the immunocomplexes were analyzed by Western blotting. We run identical membranes to identify proteins with similar molecular weight.

### Wound-healing assay

The indicated cells were cultured in 6-well plates until they reached 90% confluence, and a wound was then created with a pipette tip. After washing with PBS to remove suspended cells, the cells were cultured in a minimal medium supplemented with 5% fetal bovine serum (FBS). The same wound location was imaged at 0 and 48 h, the wound healing rate (%) was evaluated using TScratch software (Computational Science & Engineering Laboratory, Zürich, Switzerland). The experiments were repeated independently at least three times.

### RNA extraction and quantitative PCR

Total RNA was extracted using TRIzol according to the manufacturer’s protocol (Invitrogen) and the PrimeScriptTM RT reagent kit (TaKaRa, Dalian, China) was used for reverse transcription to prepare cDNA. Quantitative PCR reactions were then performed using SYBR Green chemistry using the primer sequences shown in Supplementary Table 10.

### RNA interference and transfection

Lentiviral-based short hairpin RNA (shRNA) knockdown experiments were conducted based on the pLKO.1 vector system. Control shRNA or specific sequences targeting KCTD9, ZNT9 and β-catenin in the pLKO.1 vector were transfected into HEK293T cells for 48 h using a 2:2:2:1 ratio of pLKO.1:pREV:pGag:pVSVG. Alternatively, for overexpression, the indicated cDNAs were cloned into pCDH and lentiviruses similarly prepared by transfecting HEK293T cells with a 4:3:1 ratio of pCDH:pSPDX2:PMd2G. Thereafter, the lentiviral supernatant was filtered (0.45 μm) before adding to target cells in the presence of polybrene (5 μg/ml) and selection of stably transduced cell lines with 5 μg/ml puromycin. The shRNA sequences are listed in Supplementary Table 11.

### Migration and invasion assays

For the migration assays, 2 × 10^4^ cells suspended in serum-free DMEM were seeded into the upper Transwell^™^ chambers (8.0 μm, Corning, MA, USA). Alternatively, for the invasion assays, the upper surface of the membrane was first pre-coated with Matrigel (BD Biosciences). Then, DMEM containing 10% FBS was added to the lower chambers and the cells were incubated for 24–48 h. The cells were fixed with 4% formaldehyde and the cells passing through to the lower surface of the membrane were stained with hematoxylin, imaged, and counted.

### Immunofluorescence staining (IF)

Cells were fixed with 4% formaldehyde for 30 min, permeabilized with 0.4% Triton X-100 for 20 min, washed with PBS three times, blocked with 5% bovine serum albumin (BSA) for 40 min, and incubated overnight at 4 °C with the antibodies against β-catenin (Proteintech, 1:100, 51067-2-AP). After washing three times with PBST (0.04% Tween-20 in PBS), the cells were incubated with Alexa Fluor 488-labeled Goat Anti-Rabbit IgG(H + L) (Beyotime, 1:500, A0423), washed again with PBST, and mounted. The cells were observed and recorded using epifluorescence microscopy (Zeiss).

### Luciferase and TOP-FOP Flash reporter assays

Cells seeded into 96-well culture plates were transfected with the indicated pGL3-based plasmids for 24 h before assessing reporter activity using the dual-luciferase reporter assay kit (Promega). Co-transfected Renilla luciferase measurements were used to normalize changes in transfection efficiency. Alternatively, TCF/LEF transcription activity was measured using the TOP-FOP Flash reporters purchased from Beyotime (Shanghai, China). Cells were co-transfected with a pGL6-TA plasmid (10 ng/well) and either a TOP Flash plasmid or FOP Flash plasmid (30 ng/well) for 48 h before measuring luciferase activity. The results are shown as the ratio of TOP Flash to FOP Flash activity.

### Mammalian two-hybrid assay

Complementary DNA sequences of β-catenin and ZNT9 were cloned into the pBIND and pACT vectors, respectively, and the indicated combinations of plasmids along with the pG5luc firefly luciferase vector were transfected according to the manufacturer’s instructions (Promega). Reporter activity was measured 24 h later using the dual-luciferase reporter assay kit (Promega).

### Ubiquitination assay

SW620 cells were collected and lysed in RIPA buffer at ice for 30 min, then boiled for 10 min at 95 °C, followed by sonication and dilution. After incubation at 4 °C for 30 min, samples were centrifuged at 10,000 *g* for 15 min and the supernatants were incubated with protein A/G-Sepharose beads pre-adsorbed with the indicated antibodies for 2 h at 4 °C. After washing three times using IP Buffer, proteins were eluted at 95 °C for 10 min and analyzed by Western blot.

### Xenograft and liver metastasis model

BALB/c male nude mice (aged 5 weeks and weighing 19~25 g) were randomly assigned to two groups receiving subcutaneous injections of 2.0 × 10^6^ SW620 cells into the footpad bearing either stable expression of pCDH- or pCDH-KCTD9 along with luciferase. Twenty days after injection, the mice were sacrificed, and primary tumors excised and weighed. Alternatively, a liver metastasis model was established where BALB/c nude mice were injected with 2.0 × 10^6^ cells into the spleen. After 28 days later, luciferin was injected i.p. before humanely culling the mice, and the luciferase bioluminescence of the excised tissues was measured with a Caliper IVIS Lumina II system (IVIS Spectrum; USA). All animal experiments were approved by the Animal Research Ethics Committee of the University of Science and Technology of China.

### Statistical analysis

Bioinformatics analyses were performed with R software (https://www.r-project.org/) and related R packages. Experimental data were analyzed by GraphPad Prism software. Unless stated otherwise, the data are expressed as the means±standard deviation. Statistical differences were determined by one-way analyses of variance, chi-square tests, or Student’s *t*-tests. For multiple comparisons, *P* values were adjusted using Bonferroni’s correction. Survival rates were compared by the Kaplan-Meier method and by log-rank test. A *P* value < 0.05 was considered to indicate statistical significance.

## Supplementary information

Supplementary Figure 1

Supplementary Figure 2

Supplementary Figure 3

Supplementary Figure 4

Supplementary Figure 5

Supplementary Figure 6

Supplementary Figure 7

Supplementary Figure Legends

Supplementary Table 1. Number of differentially expressed genes (DEGs) based on the RNA-seq data of three tissue samples in our hospital

Supplementary Table 2. Protein coding DEGs with consistently descending expressed trends among the adjacent normal colonic mucosa, primary colon adenocarcinoma and distant liver metastasis identified

Supplementary Table 3. List of down-regulated DEGs in colon cancer based on bioinformatic analysis of datasets

Supplementary Table 4. DEGs significantly associated with OS of TCGA-COAD data predicted by both univariable cox and log-rank methods

Supplementary Table 5. KCTD9 differential gene expression analysis in Tumor, Normal and Metastatic tissues based on TNMplot web tool

Supplementary Table 6. DEGs results based on the two groups compared in TCGA-COAD data (KCTD9 low vs. KCTD9 high in COAD)

Supplementary Table 7. Predictive results from the string database based on the protein KCTD9

Supplementary Table 8. Mass spectrometry-based experiments to identify KCTD9-interacting proteins in HCT-116 cells

Supplementary Table 9. clinical synopsis of the patients' cohort used in the study

Supplementary Table 10. Primer of QRT PCR

Supplementary Table 11. shRNA sequences

Original Data File

aj-checklist

## Data Availability

All data generated and analyzed during this study are included in this published article and its additional file.
