# Supplementary figures and images for: KCTD9 inhibits the Wnt/β-catenin pathway by decreasing the level of β-catenin in colorectal cancer
Source: Cell Death Dis. 2022 Sep 2;13(9):761. doi: 10.1038/s41419-022-05200-1 (PMC9440223; doi:10.1038/s41419-022-05200-1)

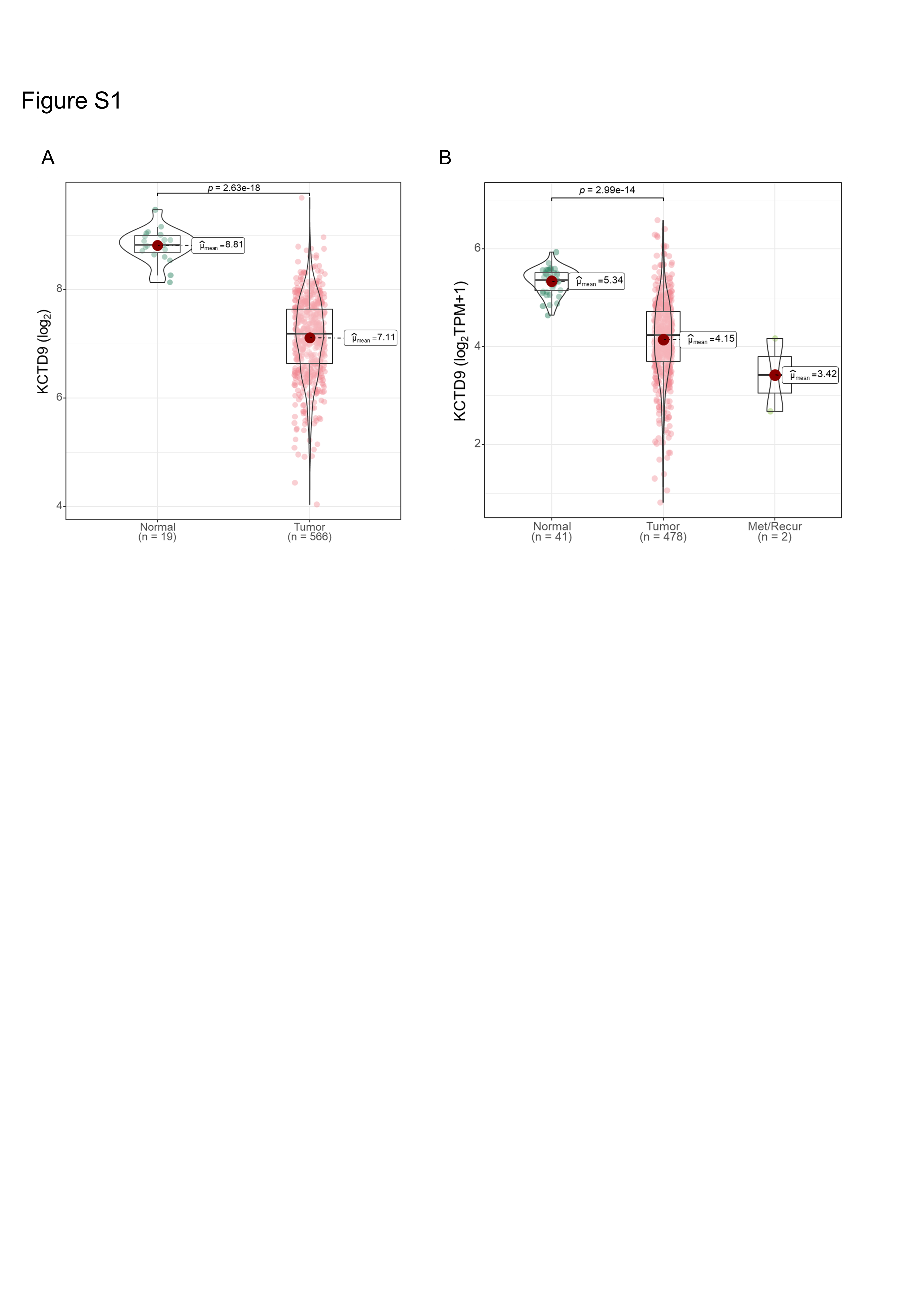

Supplement: Supplementary file 1 — Supplementary Figure 1 [file 41419_2022_5200_MOESM1_ESM.png]

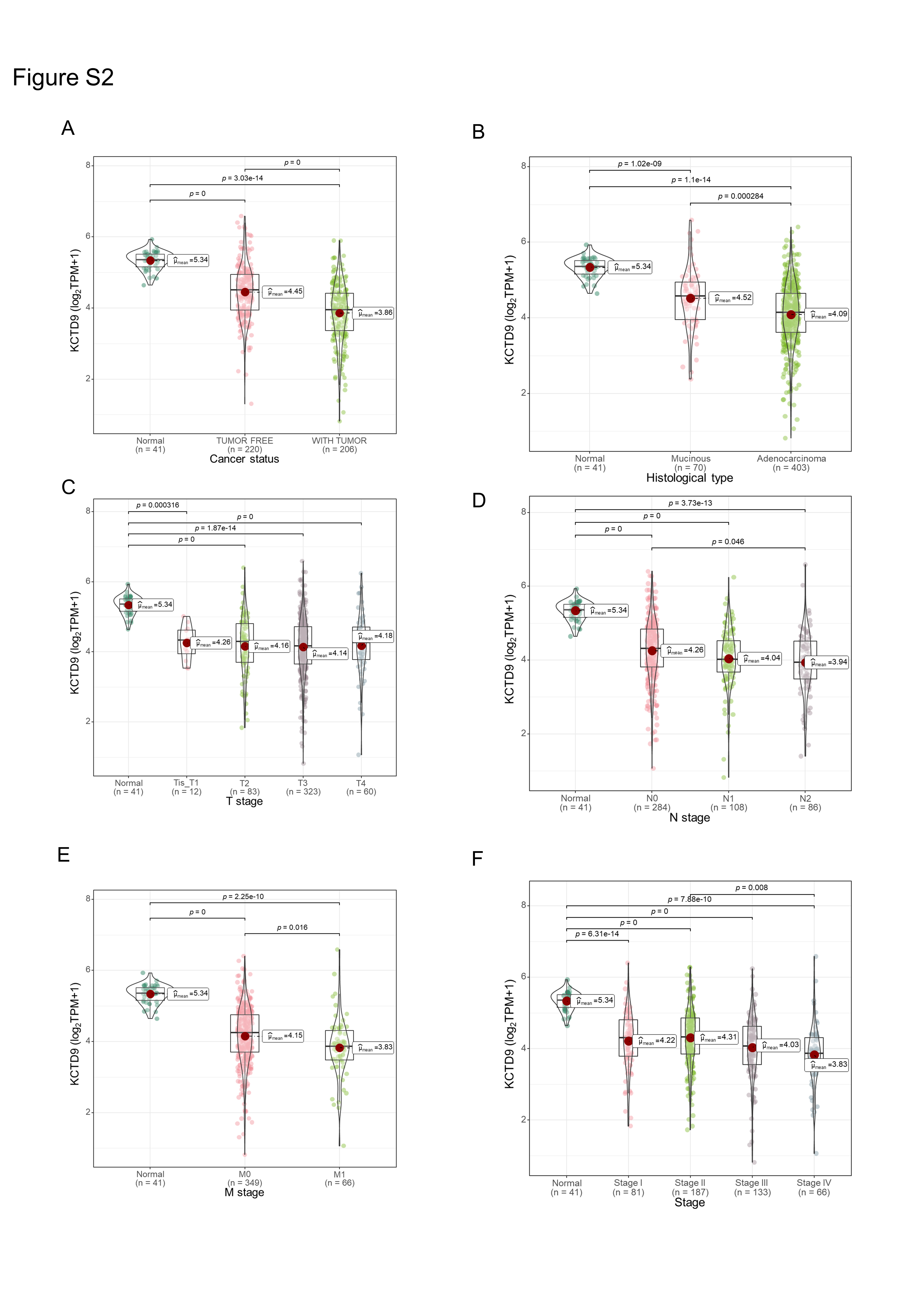

Supplement: Supplementary file 2 — Supplementary Figure 2 [file 41419_2022_5200_MOESM2_ESM.png]

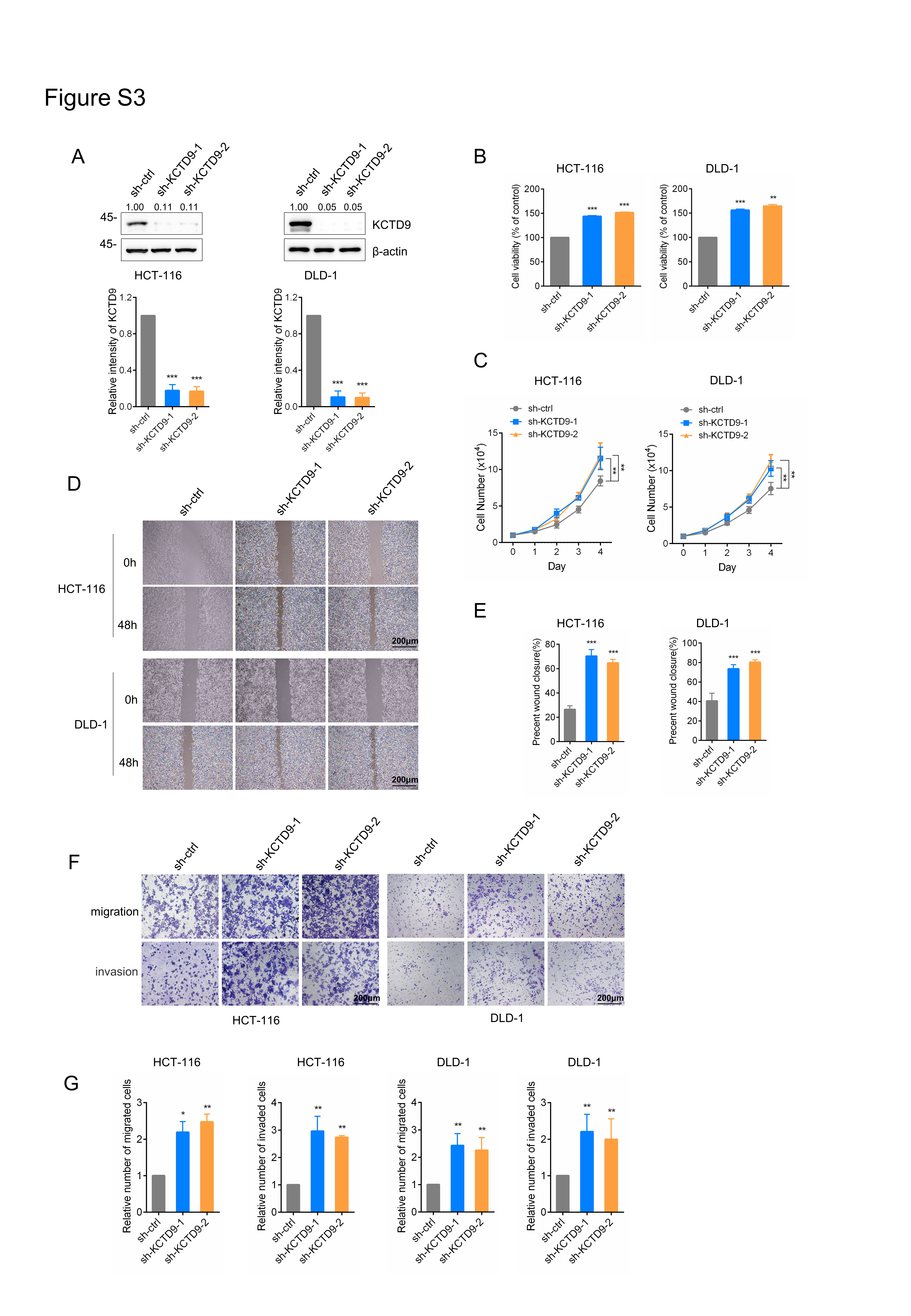

Supplement: Supplementary file 3 — Supplementary Figure 3 [file 41419_2022_5200_MOESM3_ESM.png]

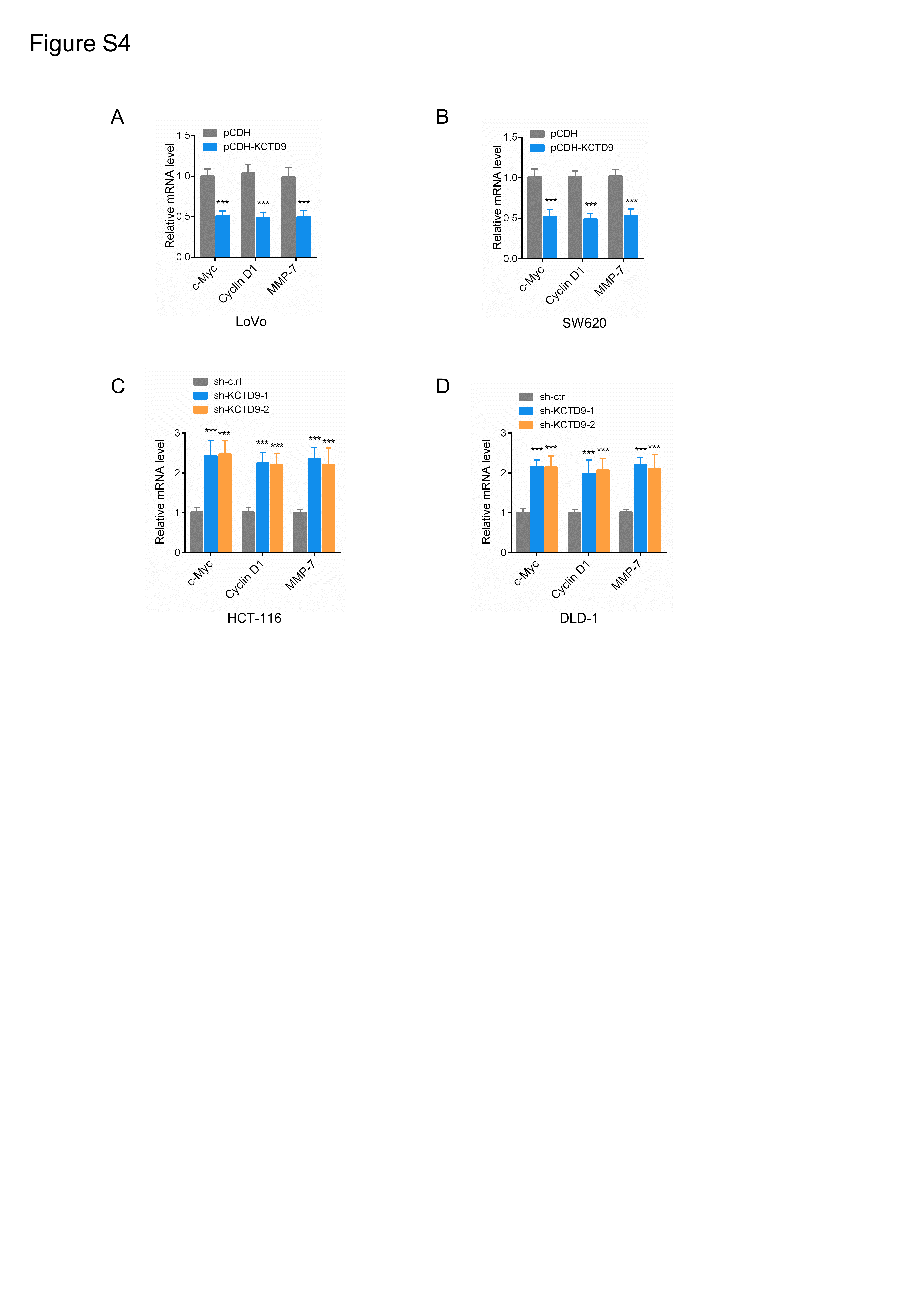

Supplement: Supplementary file 4 — Supplementary Figure 4 [file 41419_2022_5200_MOESM4_ESM.png]

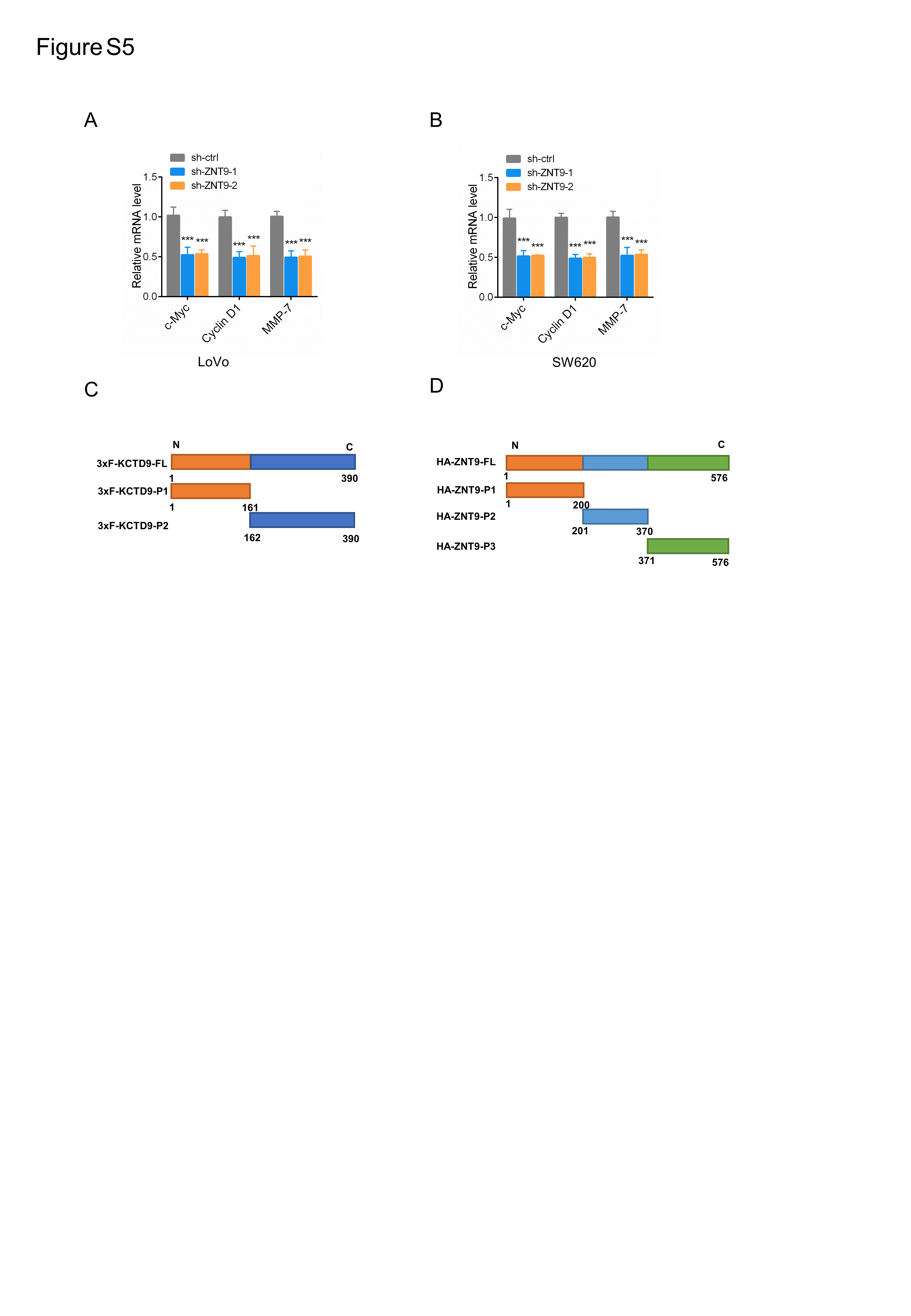

Supplement: Supplementary file 5 — Supplementary Figure 5 [file 41419_2022_5200_MOESM5_ESM.png]

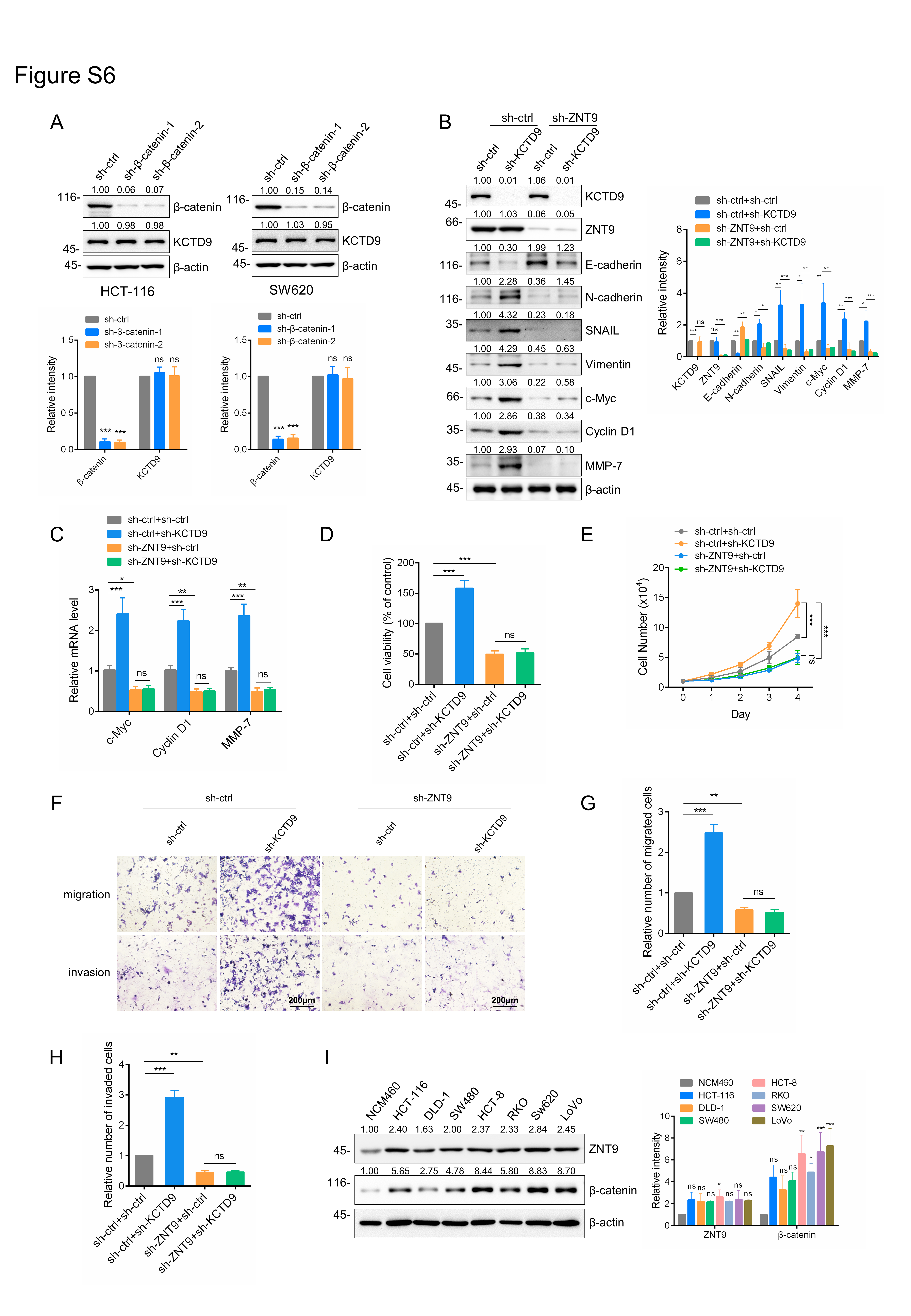

Supplement: Supplementary file 6 — Supplementary Figure 6 [file 41419_2022_5200_MOESM6_ESM.png]

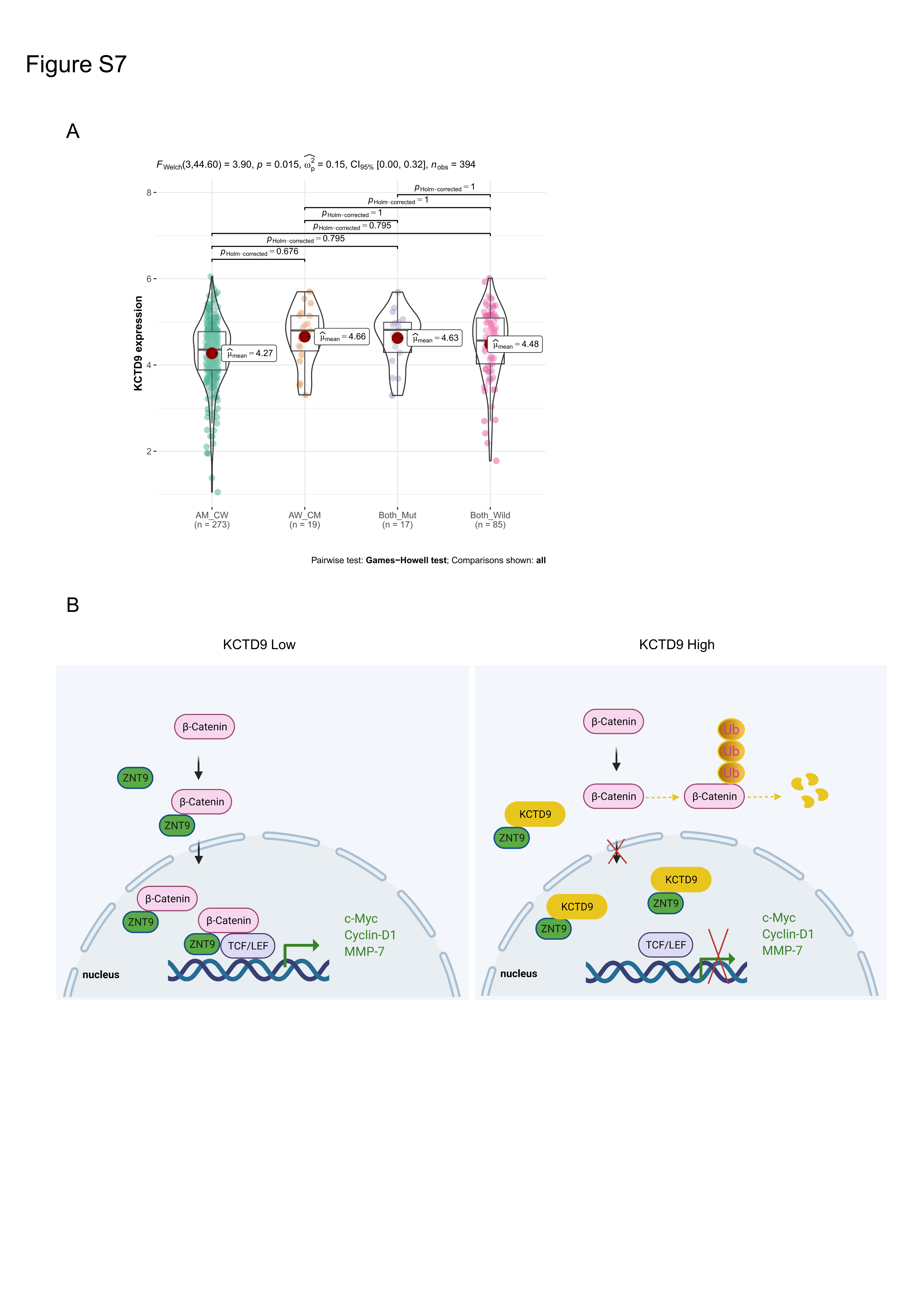

Supplement: Supplementary file 7 — Supplementary Figure 7 [file 41419_2022_5200_MOESM7_ESM.png]
