# Supplementary material for: KCTD9 inhibits the Wnt/β-catenin pathway by decreasing the level of β-catenin in colorectal cancer
Source: Cell Death Dis. 2022 Sep 2;13(9):761. doi: 10.1038/s41419-022-05200-1 (PMC9440223; doi:10.1038/s41419-022-05200-1)
Supplement: Supplementary file 8 — Supplementary Figure Legends [file 41419_2022_5200_MOESM8_ESM.docx]

Supplementary Figure Legends

Fig. S1. Supplementary data related to Figure 1

(A, B) Comparison of KCTD9 mRNA expression in normal, CRC, and metastatic / recurrent tissues based on the GSE39582 (A) and TCGA-COAD (B) datasets.

Fig. S2. Supplementary data related to Figure 2

(A-F) Comparison of KCTD9 expression levels in patient tissues from the TCGA-COAD dataset stratified according to cancer status (A), histological type (B), tumor (T) stage (C), lymph node (N) stage (D), metastasis (M) stage (E) and overall TNM stage (F).

Fig. S3. Supplementary data related to Figure 3

(A) Western blotting analysis of the CRC cell lines HCT-116 and DLD-1 after transduction with lentiviral particles based on control shRNA (sh-ctrl) or vectors containing independent sequences targeting KCTD9 (sh-KCTD9-1 and KCTD9-2). β-actin was used as a loading control. Data shown represent three independent experiments (top) and relative density were quantified by using ImageJ software (bottom).

(B) Assessment of cell viability in the cells from (A) measured using CCK-8 assays.

(C) Assessment of proliferation in the cells from (A) measured using Count Star (Countstar BioTech) over 1-4 days by cell counting.

(D, E) Assessment of migration in the cells from (A) measured in wound healing assays. Representative images at time 0 and 48 h (scale bar =200 µm) (D) with quantitation of wound closure (E). (D) represent three independent experiments.

(F, G) Assessment of migration and invasion in the cells from (A) using Transwell™ assays. Representative images of migrating/invading cells after 24 h (scale bar =200 µm) (F) with comparative quantitation of total migrating cells (G). (F) represent three independent experiments.

(A, B, E and G) are mean ± SD; n=3 independent experiments, one-way ANOVA with Tukey’s multiple comparison post-test, *p < 0.05, **p < 0.01, ***p < 0.001. (C) is mean ± SD, n=3 independent experiments, two-way ANOVA with Bonferroni’s multiple comparison post-test, ns, not significant, **p < 0.01.

Fig. S4. Supplementary data related to Figure 4

(A-D) Comparative analysis of c-Myc, cyclin D1, and MMP-7 mRNA levels measured using qRT-PCR in LoVo (A) or SW620 (B) cells after overexpression of KCTD9 or in HCT-116 (C) or DLD-1 (D) cells after knockdown of KCTD9. Data shown mean ± SD, n=3 independent experiments, two-way ANOVA with Bonferroni’s multiple comparison post-test, ***p < 0.001.

Fig. S5. Supplementary data related to Figure 5

(A, B) Comparative analysis of c-Myc, cyclin D1, and MMP-7 mRNA levels measured using qRT-PCR in LoVo (A) and SW620 (B) cells after knockdown of ZNT9. Data shown mean ± SD, n=3 independent experiments, two-way ANOVA with Bonferroni’s multiple comparison post-test, ***p < 0.001.

(C, D) Schematic showing the functional domain organization of KCTD9 (C) and ZNT9 (D), respectively. ZNT9 and KCTD9 domain truncation mutants were constructed using HA and Flag epitope tags, respectively, according to the design shown under the full length (FL) construct. Numbers indicate the amino acid positions.

Fig. S6. Supplementary data related to Figure 6

(A) Western blot analysis of the levels of KCTD9 and β-catenin in HCT-116 and SW620 cells infected with control (sh-ctrl) lentiviruses or two independent shRNAs targeting β-catenin (sh-β-catenin-1 and sh-β-catenin-2). β-actin was used as a loading control throughout. Data shown represent three independent experiments (top) and relative density were quantified by using ImageJ software (bottom).

(B) Western blot comparing the levels of KCTD9, β-catenin, E-cadherin, N-cadherin, SNAIL, vimentin, c-Myc, cyclin D1, and MMP-7 in SW620 cells infected with the indicated combinations of sh-ctrl, sh-KCTD9, and sh-ZNT9 lentiviruses. Data shown represent three independent experiments (left) and relative density were quantified by using ImageJ software (right).

(C) qPCR analysis of samples from (B) comparing the mRNA levels of c-Myc, cyclin D1, and MMP-7.

(D) Assessment of cell viability in the cells from (B) measured using CCK-8 assays.

(E) Assessment of proliferation in the cells from (B) measured using Count Star (Countstar BioTech) over 1-4 days by cell counting.

(F-H) Assessment of migration and invasion in the cells from (B) using Transwell™ assays. Representative images of migrating/invading cells after 24 h (scale bar =200 µm) (F) with comparative quantitation of total migrating (G) or invading (H) cells. (F) represent three independent experiments.

(I) Comparative analysis of β-catenin and ZNT9 expression in the normal human colon mucosal epithelial cell line NCM460 and a panel of CRC-derived cell lines using Western blotting. Data shown represent three independent experiments (left) and relative density were quantified by using ImageJ software (right).

(A, B, D, G, H and I) are mean ± SD; n=3 independent experiments, one-way ANOVA with Tukey’s multiple comparison post-test, ns, not significant, *p < 0.05, **p < 0.01, ***p < 0.001. (C and E) are mean ± SD, n=3 independent experiments, two-way ANOVA with Bonferroni’s multiple comparison post-test, ns, not significant, *p < 0.05, **p < 0.01, ***p < 0.001.

**Fig. S7. Supplementary data related to Figure 7**

(A) *KCTD9* mRNA expression levels based on the mutation status of *APC* and *CTNNB1* genes in the TCGA COAD dataset. MAF files and corresponding clinical information for a total of 394 COAD cases were downloaded from the TCGA dataset (<https://portal.gdc.cancer.gov/>). Based on the mutation status of *APC* and *CTNNB1* genes, the cases were divided into four groups (AM_CW, *APC* mutant and *CTNNB1* wildtype (n =273); AW_CM, *APC* wildtype and *CTNNB1* mutant group (n =19); Both Mut, both *APC* and *CTNNB1* mutant (n =17); Both Wild, both *APC* and *CTNNB1* wildtype (n =85) and comparative KCTD9 mRNA expression levels analyzed using R software and ggstatsplot package. There was no statistical difference of KCTD9 mRNA expression levels among the four groups.

(B) Working model diagram delineating the role of KCTD9 in the Wnt/β-catenin signaling pathway in CRC. The model was created with BioRender.com (2020) (https://app.biorender.com/biorender-templates).
