# Supplementary material for: KCTD9 inhibits the Wnt/β-catenin pathway by decreasing the level of β-catenin in colorectal cancer
Source: Cell Death Dis. 2022 Sep 2;13(9):761. doi: 10.1038/s41419-022-05200-1 (PMC9440223; doi:10.1038/s41419-022-05200-1)

Figure 1C

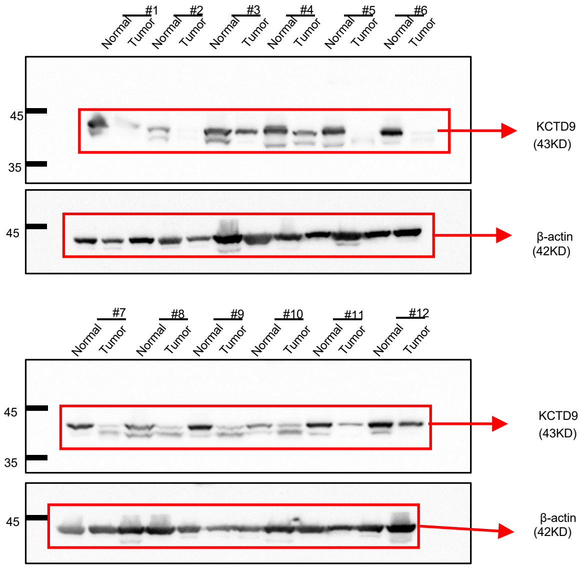

Figure 1G-1

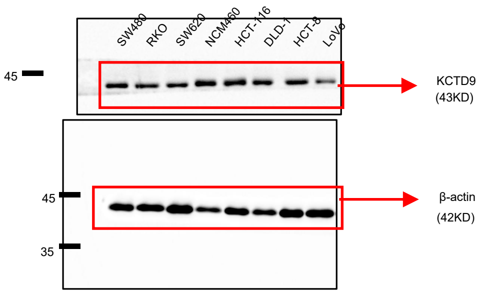

Figure 1G-2

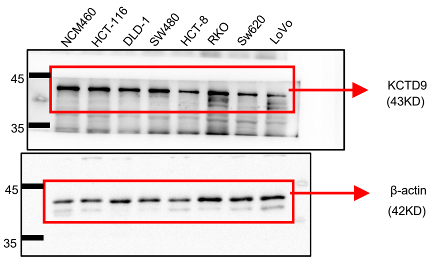

Figure 1G-3

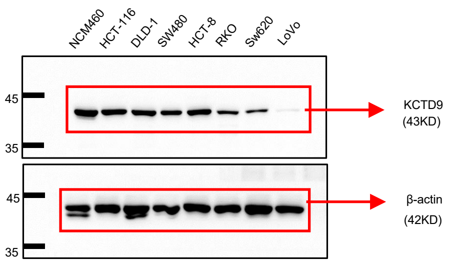

Figure 3A-1

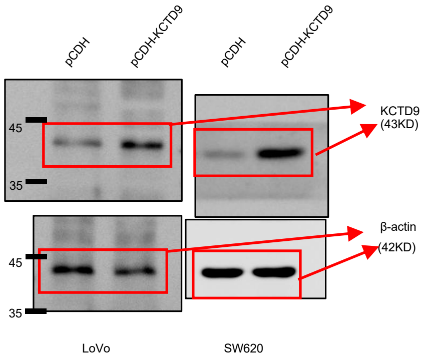

Figure 3A-2

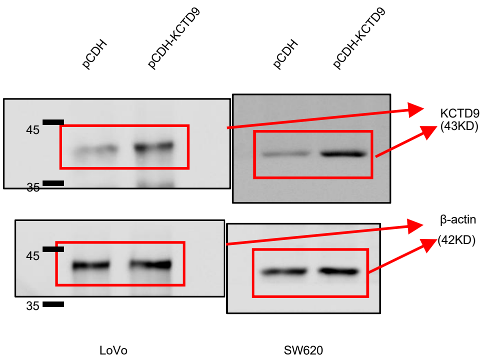

Figure 3A-3

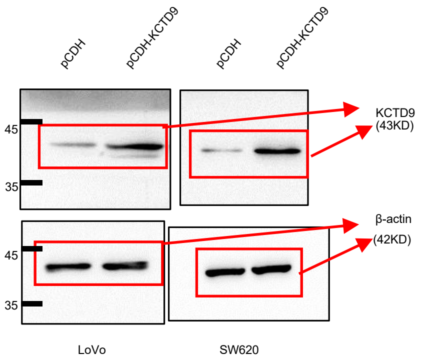

Figure 4A-1

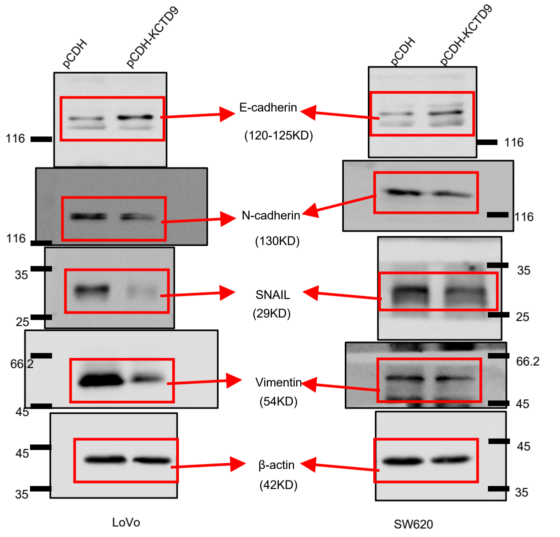

Figure 4B-1

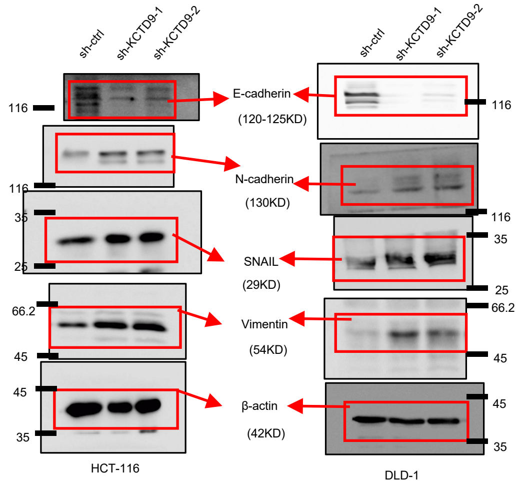

Figure 4A-2

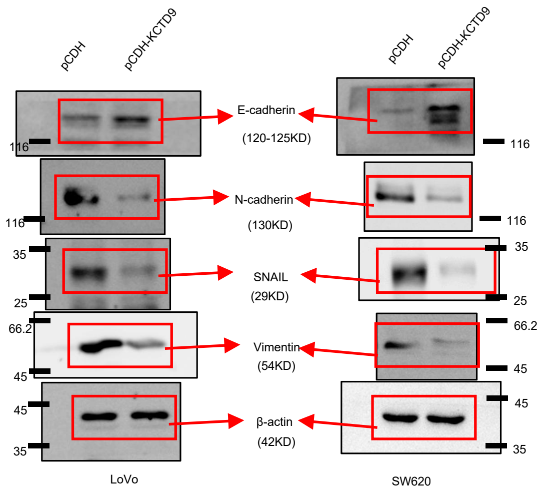

Figure 4B-2

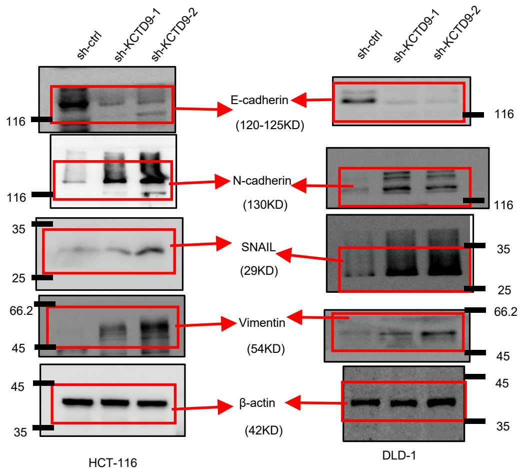

Figure 4A-3

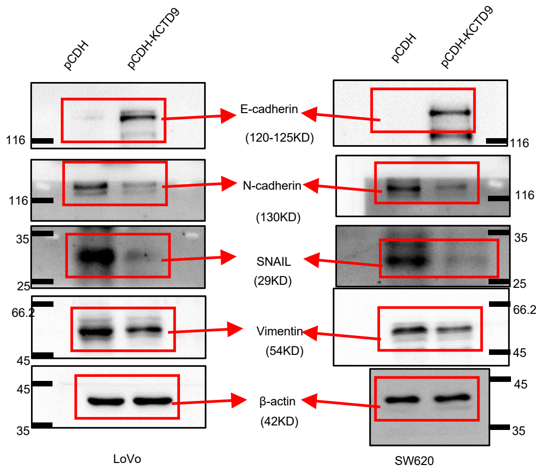

Figure 4B-3

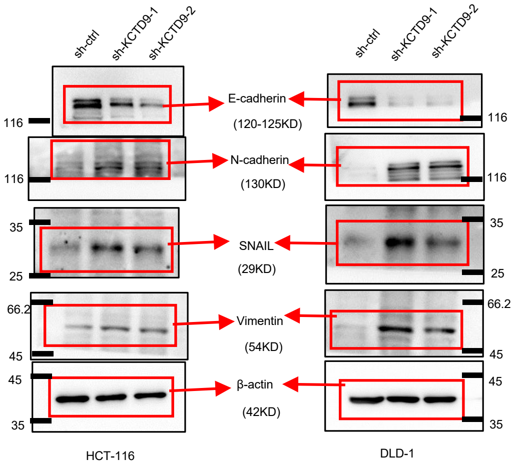

Figure 4E-1

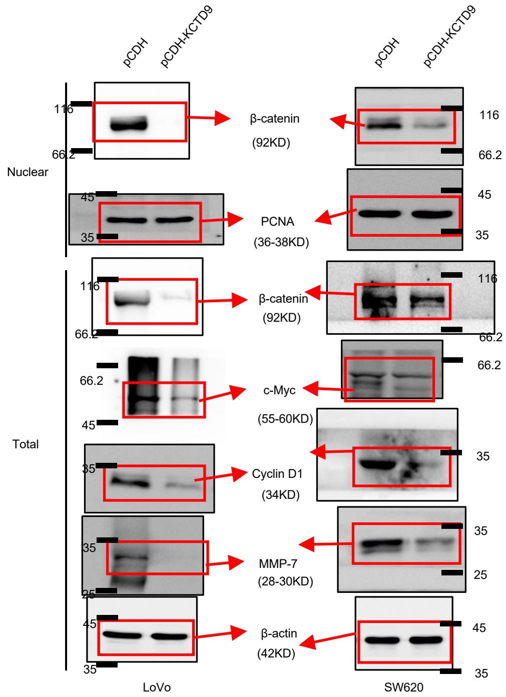

Figure 4E-2

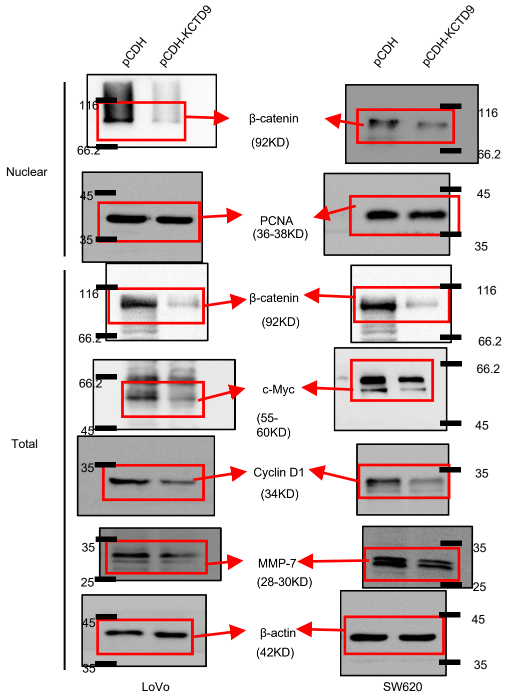

Figure 4E-3

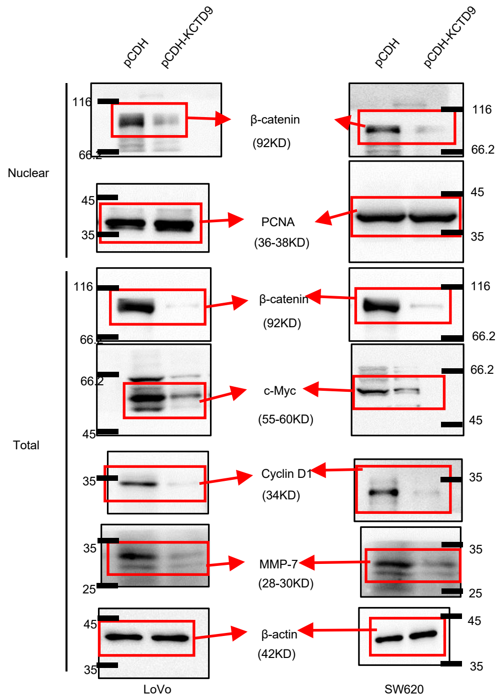

Figure 4F-1

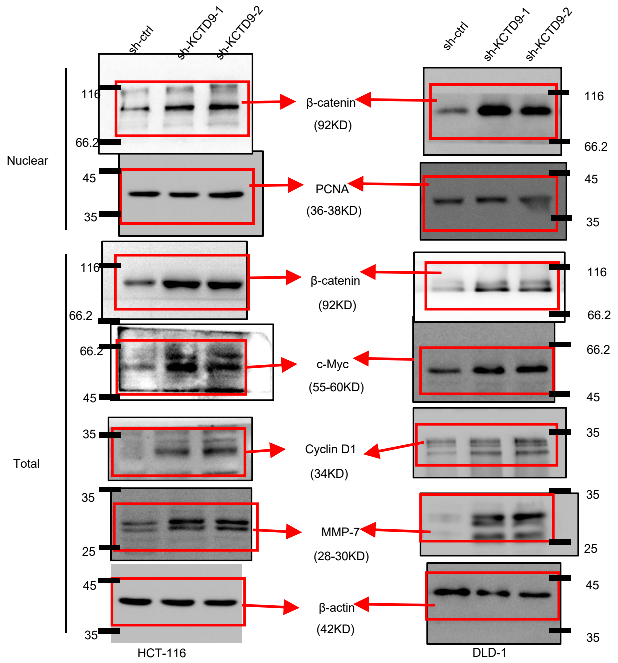

Figure 4F-2

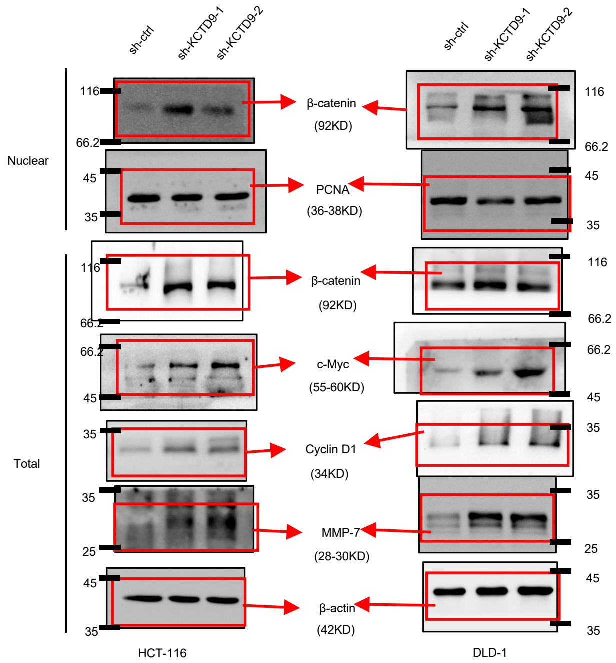

Figure 4F-3

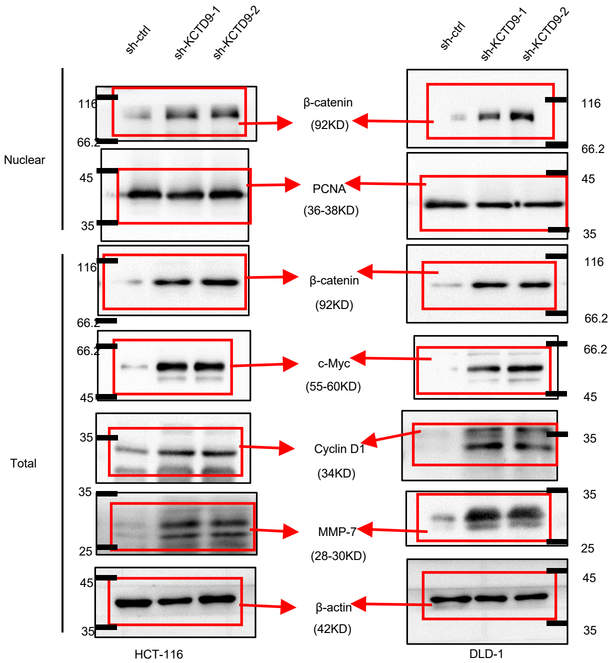

Figure 5D-1

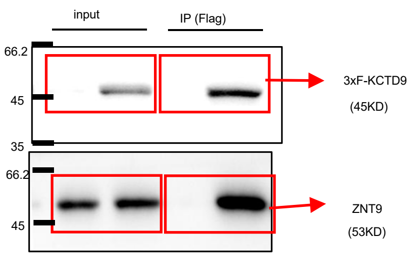

Figure 5D-2

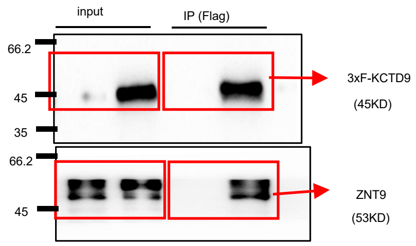

Figure 5D-3

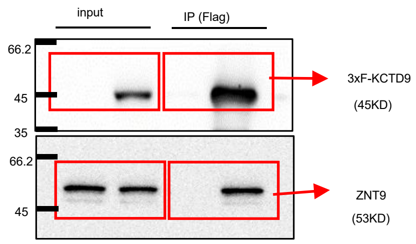

Figure 5E-1

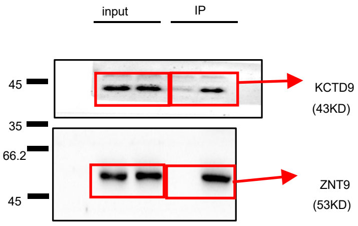

Figure 5E-2

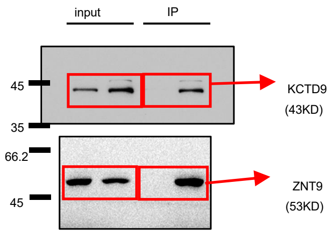

Figure 5E-3

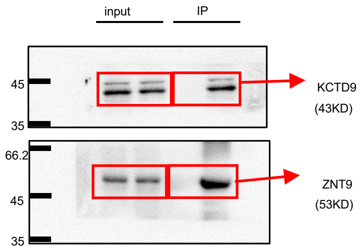

Figure 5F-1

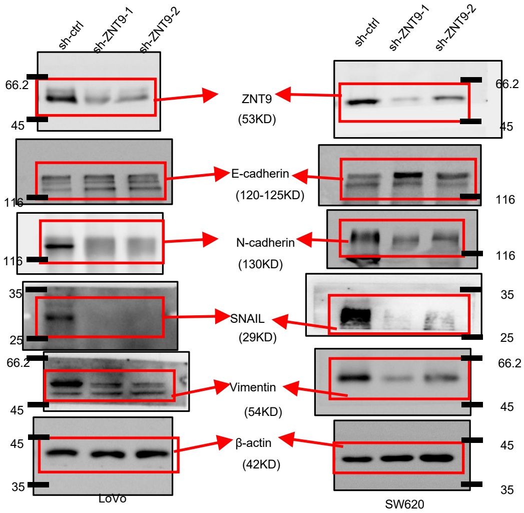

Figure 5F-2

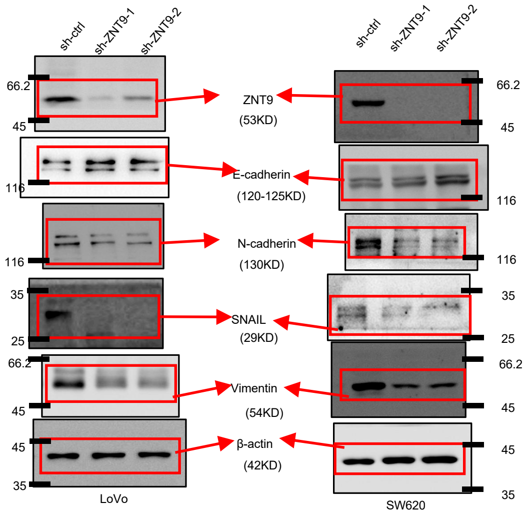

Figure 5F-3

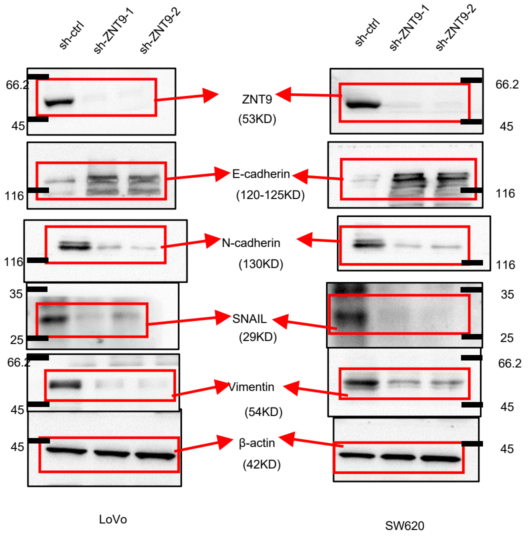

Figure 5G-1

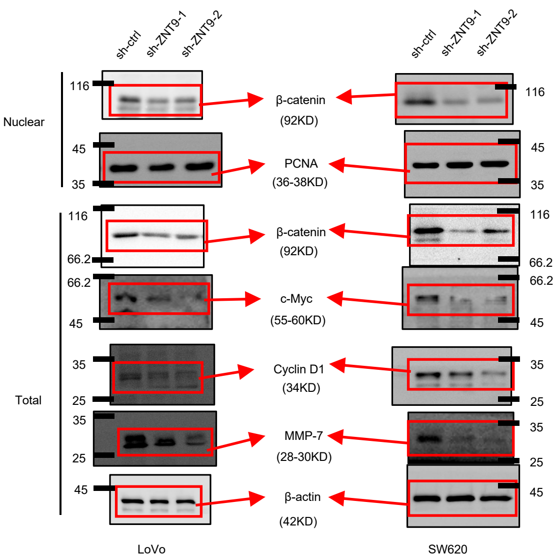

Figure 5G-2

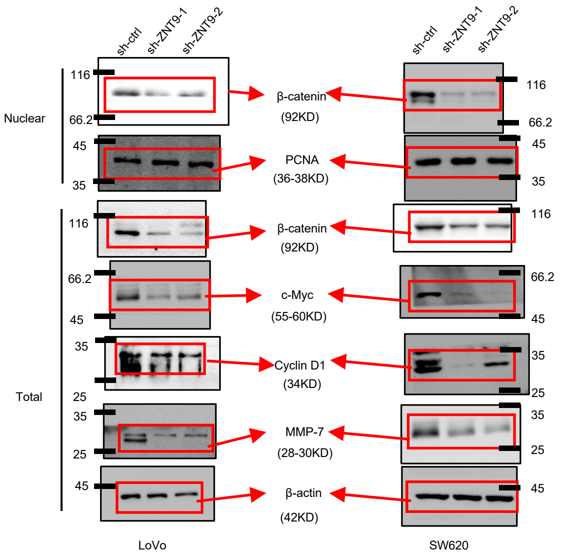

Figure 5G-3

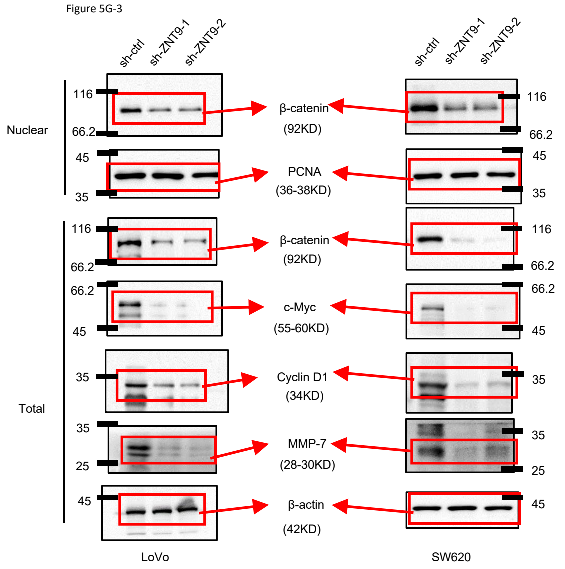

Figure 5H-1

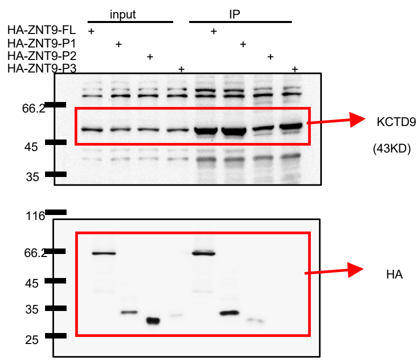

Figure 5H-2

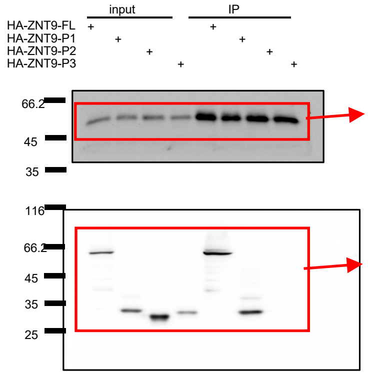

Figure 5H-3

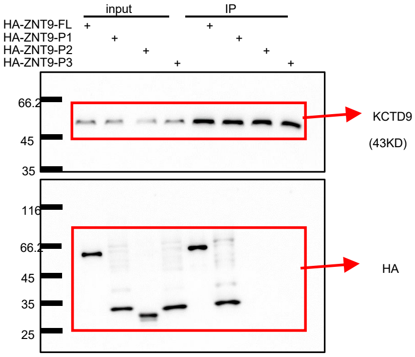

Figure 5I-1

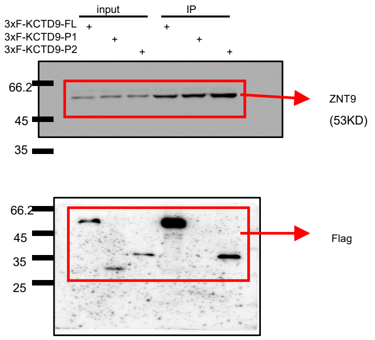

Figure 5I-2

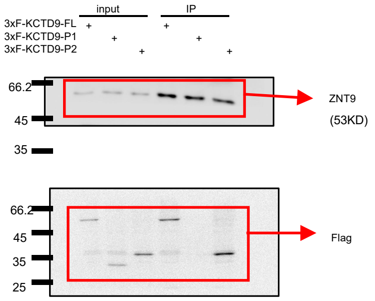

Figure 5I-3

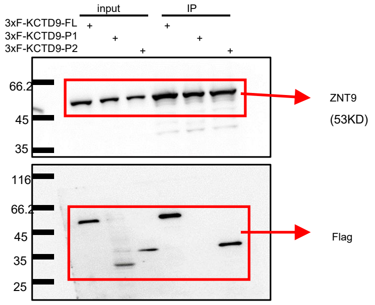

Figure 5J-1

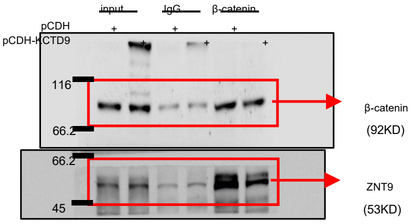

Figure 5J-2

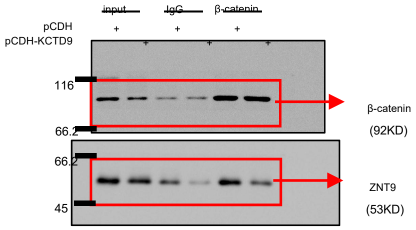

Figure 5J-3

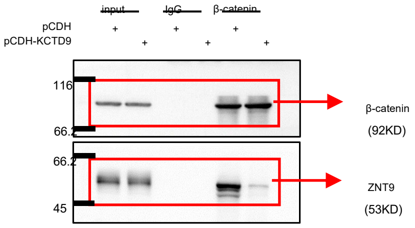

Figure 5K-1

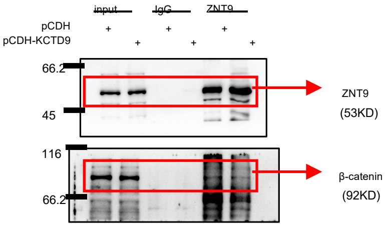

Figure 5K-2

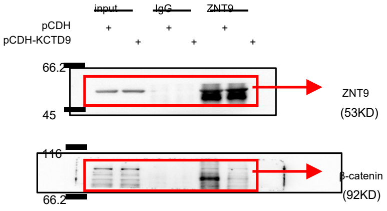

Figure 5K-3

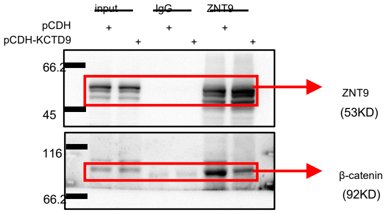

Figure 5L-1

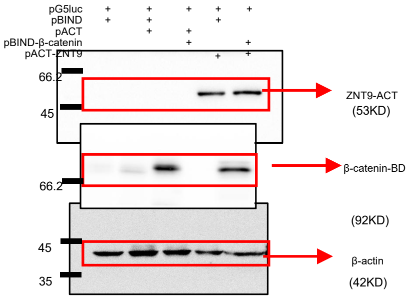

Figure 5L-2

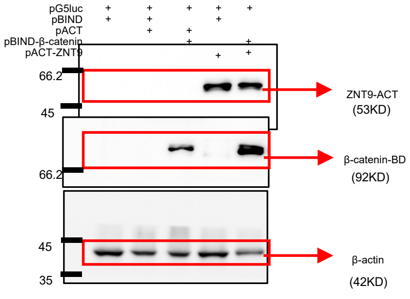

Figure 5L-3

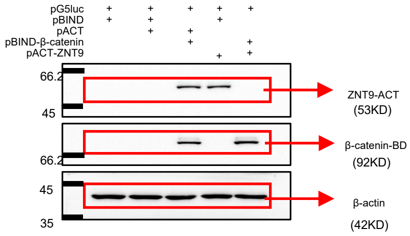

Figure 6A-1

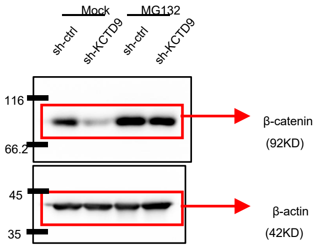

Figure 6A-2

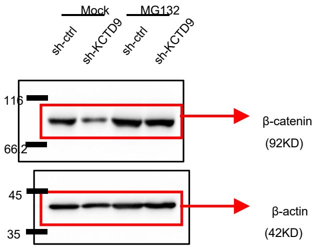

Figure 6A-3

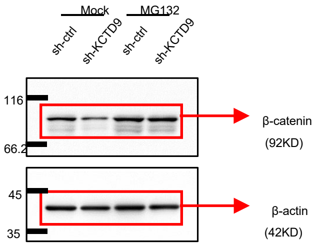

Figure 6B-1

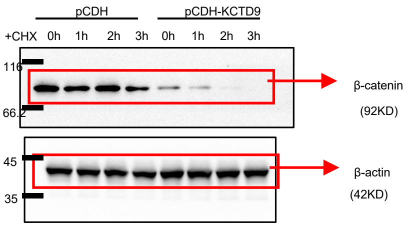

Figure 6B-2

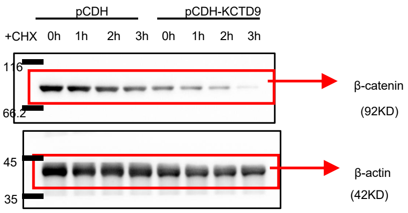

Figure 6B-3

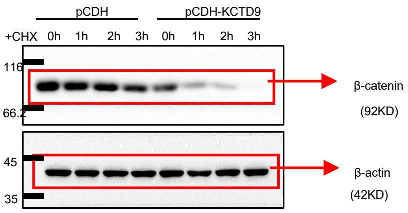

Figure 6D-1

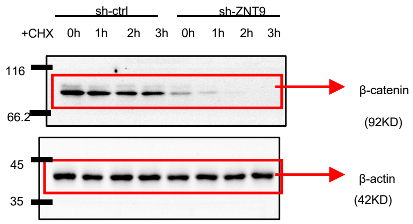

Figure 6D-2

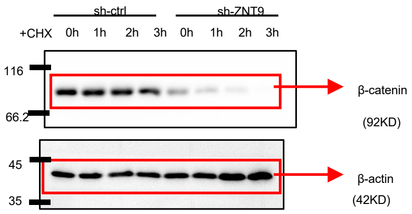

Figure 6D-3

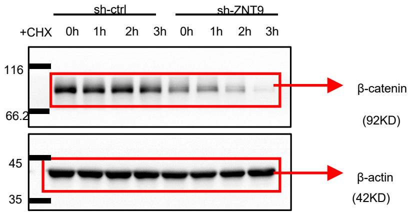

Figure 6F-1

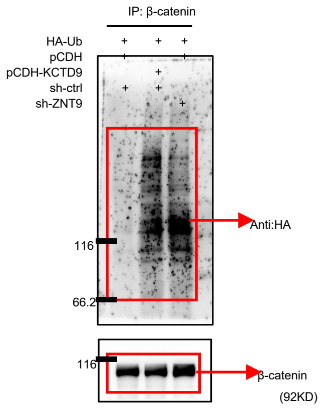

Figure 6F-2

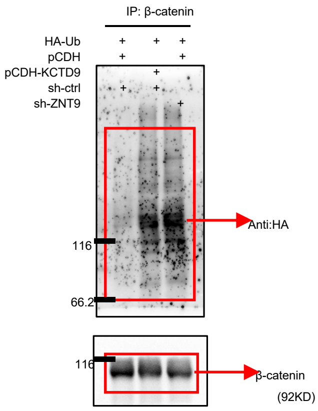

Figure 6F-3

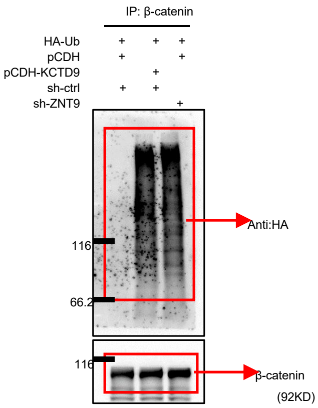

Figure 6G-1

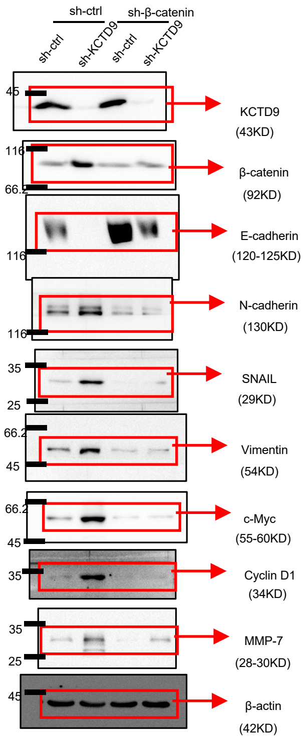

Figure 6G-2

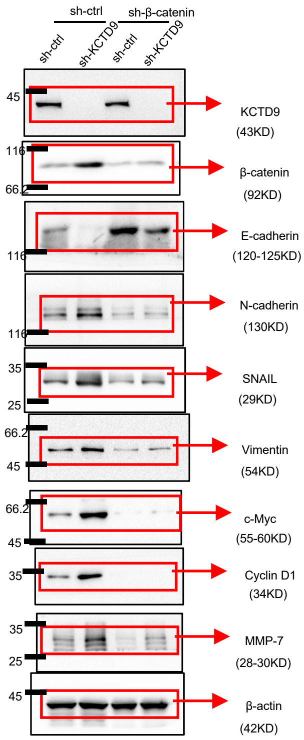

Figure 6G-3

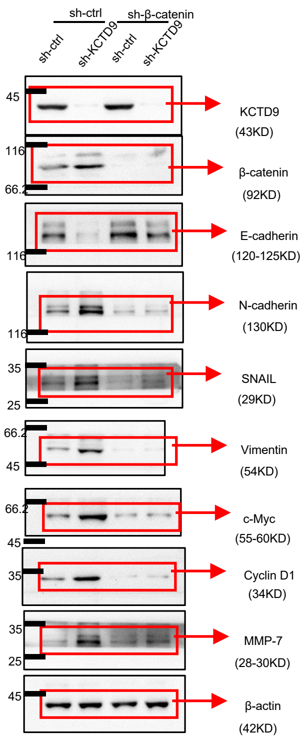

Figure S3A-1

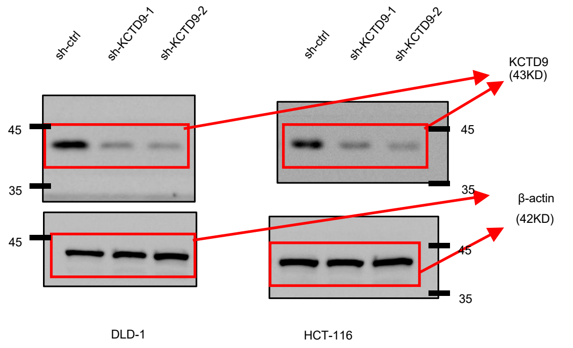

Figure S3A-2

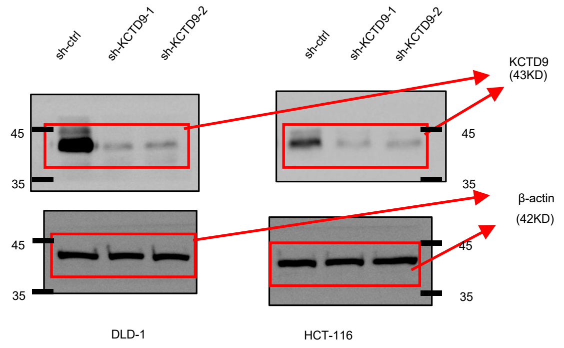

Figure S3A-3

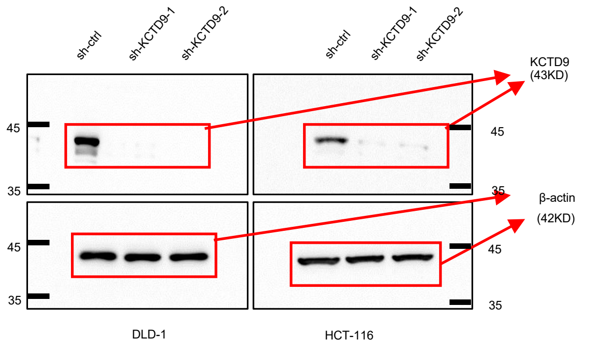

Figure S6A-1

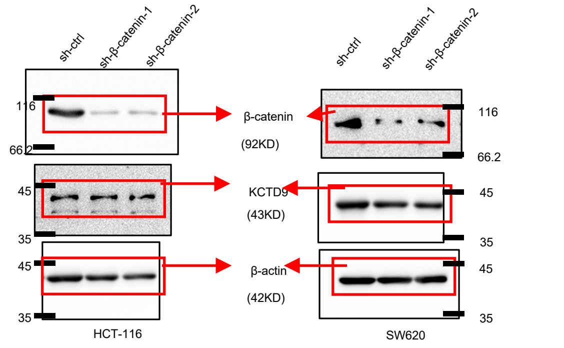

Figure S6A-2

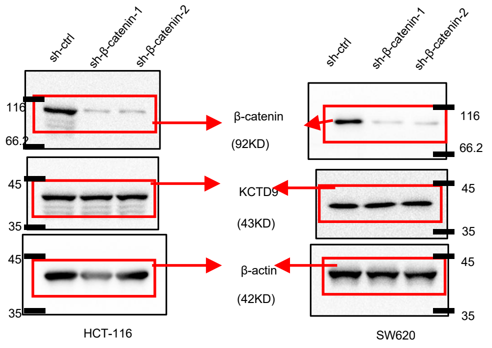

Figure S6A-3

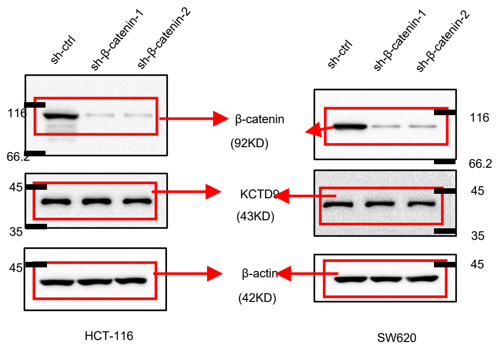

Figure S6B-1

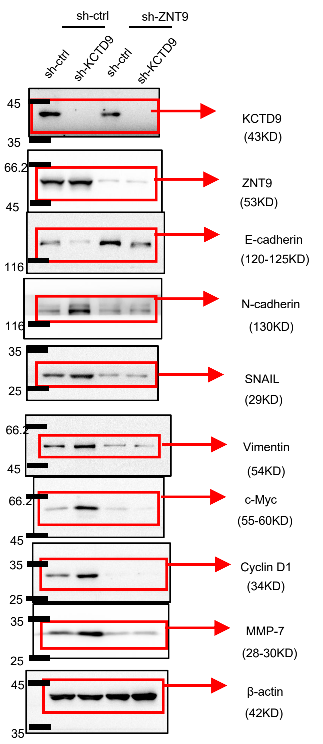

Figure S6B-2

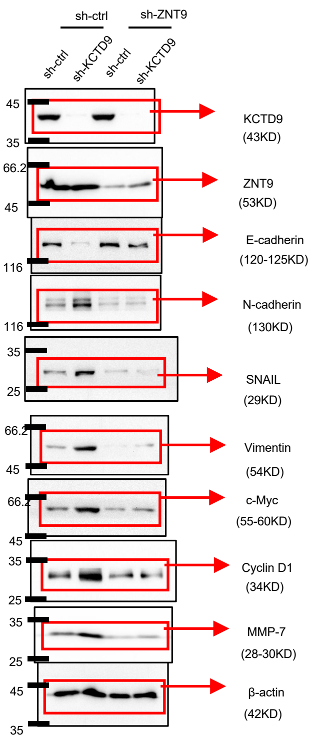

Figure S6B-3

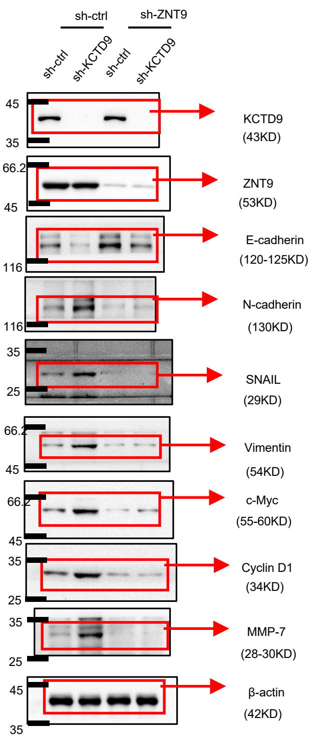

Figure S6I-1

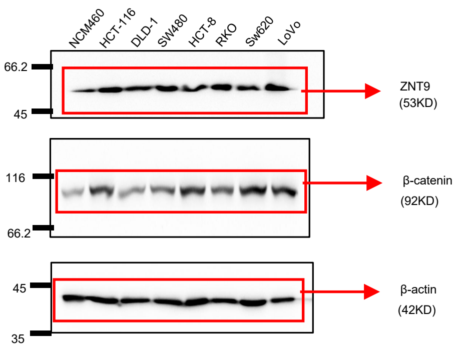

Figure S6I-2

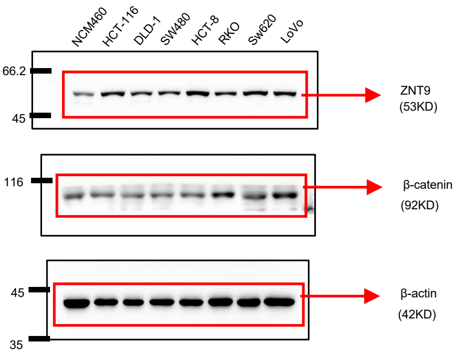

Figure S6I-3

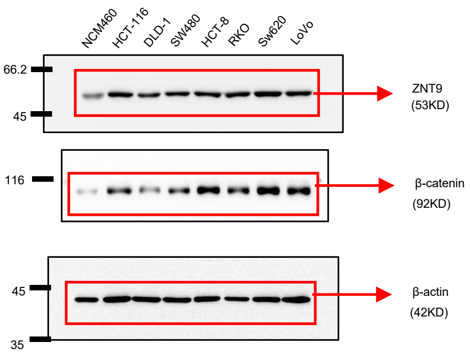

Supplement: Supplementary file 20 — Original Data File [file 41419_2022_5200_MOESM20_ESM.pdf]
